# Long-read assembled metagenomic approaches improve our understanding on metabolic potentials of microbial community in mangrove sediments

**DOI:** 10.1186/s40168-023-01630-x

**Published:** 2023-08-23

**Authors:** Zhi-Feng Zhang, Li-Rui Liu, Yue-Ping Pan, Jie Pan, Meng Li

**Affiliations:** 1 Archaeal Biology Center, Institute for Advanced Study, Shenzhen University, Shenzhen, China; 2 Shenzhen Key Laboratory of Marine Microbiome Engineering, Institute for Advanced Study, Shenzhen University, Shenzhen, China; 3 Present Address: Southern Marine Science and Engineering Guangdong Laboratory (Guangzhou), Guangzhou, 511458 China

**Keywords:** PacBio SMRT sequencing, Metagenome-assembled genomes, Microbial community, Metabolic potentials, New bacterial phylum, Mangrove sediment

## Abstract

**Background:**

Mangrove wetlands are coastal ecosystems with important ecological features and provide habitats for diverse microorganisms with key roles in nutrient and biogeochemical cycling. However, the overall metabolic potentials and ecological roles of microbial community in mangrove sediment are remained unanswered. In current study, the microbial and metabolic profiles of prokaryotic and fungal communities in mangrove sediments were investigated using metagenomic analysis based on PacBio single-molecule real time (SMRT) and Illumina sequencing techniques.

**Results:**

Comparing to Illumina short reads, the incorporation of PacBio long reads significantly contributed to more contiguous assemblies, yielded more than doubled high-quality metagenome-assembled genomes (MAGs), and improved the novelty of the MAGs. Further metabolic reconstruction for recovered MAGs showed that prokaryotes potentially played an essential role in carbon cycling in mangrove sediment, displaying versatile metabolic potential for degrading organic carbons, fermentation, autotrophy, and carbon fixation. Mangrove fungi also functioned as a player in carbon cycling, potentially involved in the degradation of various carbohydrate and peptide substrates. Notably, a new candidate bacterial phylum named as *Candidatus* Cosmopoliota with a ubiquitous distribution is proposed. Genomic analysis revealed that this new phylum is capable of utilizing various types of organic substrates, anaerobic fermentation, and carbon fixation with the Wood-Ljungdahl (WL) pathway and the reverse tricarboxylic acid (rTCA) cycle.

**Conclusions:**

The study not only highlights the advantages of HiSeq-PacBio Hybrid assembly for a more complete profiling of environmental microbiomes but also expands our understanding of the microbial diversity and potential roles of distinct microbial groups in biogeochemical cycling in mangrove sediment.

Video Abstract

**Supplementary Information:**

The online version contains supplementary material available at 10.1186/s40168-023-01630-x.

## Introduction

With the development of high-throughput sequencing techniques, metagenomic sequencing has becoming a paradigm shift for the study and exploration on microbial community [1, 2]. Contrast to the cultivation bottleneck that limits our view and appreciation of the microbial world, metagenomics provides us a relatively unbiased view of not only the structure but also the metabolic potential of a community [1, 3]. This culture-independent technique based on shotgun sequencing has been applied in a broad field of microbiology [4], including clinical microbiology, environmental microbiology, and so on [5–7]. In recent years, metagenomic assembling and binning enabled the direct recovery of individual genomes from complex environmental microbiomes and have greatly improved our understanding on the function and evolution of the microbial dark matter [2, 8].

Illumina sequencing platform has become the most widely used method for metagenomic studies because of its high accuracy (0.1–1% error rates) and throughput [9]. However, Illumina short-read sequences often result in highly fragmented genomes when performing de novo assemblies for environmental samples and pure cultures, since short reads fail to correctly assemble genomic regions containing longer repetitive elements [4, 10]. This fragmentation problem is magnified due to the existence of intergenomic repeats, especially when sequenced microbial communities contain closely related species or subspecies in different and unknown abundances [4, 11, 12].

Represented by Pacific Biosciences (PacBio) and Oxford Nanopore Technologies (ONT), the recently emerged third-generation sequencing platforms offer a possible solution to partly resolve ambiguous repetitive regions and to improve genome contiguity [10, 13]. Although these platforms are criticized due to its considerably high error rate (> 10%), the produced long reads (up to 10–12 kb of mean read length) can generate genomes with high degree of completeness [9, 14, 15]. Studies based on mock microbial community revealed that Hybrid assembly using both short and long reads (either ONT or PacBio) greatly improves the contiguity of assembly with high accuracy reaching ~ 99.4–99.8% of the assembly accuracy) using Illumina short reads alone [4, 16]. In this context, PacBio and ONT sequencing have been more frequently adopted in recent metagenomic studies [14, 15, 17–20]. For instance, by using the HiSeq-PacBio Hybrid metagenomic sequencing approach, Jin et al. retrieved 475 high-quality MAGs from 12 fecal samples, 234 of which were currently uncultured and 24 were newly found [17]. Besides, Somerville et al. demonstrated a de novo assembly of complete genomes of all dominant strains, some bacterial plasmids, and phages and a corresponding prophage from low-complexity metagenome samples using Illumina and PacBio Hybrid assembly [21]. However, the HiSeq-PacBio Hybrid metagenomic approach has been rarely used to investigate environmental microbiomes of natural habitats, such as mangrove wetlands.

Mangrove wetlands represent an important coastal ecotype widely distributed in tropical and subtropical regions [22, 23]. Because of the high productivity and great contribution of organic carbon to the ocean, mangroves are known as “blue carbon sink,” despite the relatively low covering area [24, 25]. Mangrove ecosystems are characterized by specific ecological features, such as high nutrient concentration, high salinity, low oxygen and pH, and strong redox potential, providing habitat for numerous adapted organisms, especially microorganisms [26, 27]. A variety of metagenomic research adopting Illumina sequencing have been performed to study the microbial community in mangroves, with an initial focus on the overall metabolic potentials or specific metabolic pathways of microbial communities in mangrove sediments [28–32]. Later, MAGs of some microbial groups with particular metabolic potentials have been reconstructed from mangrove sediment metagenomes, such as Bathyarchaeota in aromatic compound degradation, porphyrin biosynthesis, and urea utilization [33, 34], Methanofastidiosa in methanogenesis [35], and Gerdarchaeota in organic matter degradation [36]. Overall, metagenomic studies using Illumina sequencing preliminarily uncovered the important roles of microorganisms in driving complex nutrient and biogeochemical cycling by various metabolic pathways [37, 38], such as ammonia oxidation [39, 40], organic carbon degradation [33, 36], methane metabolism [35, 41], and sulfate reduction [34, 42]. However, due to the limitation of Illumina short reads discussed above, microbial diversity in mangrove sediments remains largely undiscovered. In addition, previous studies mostly focused on prokaryotic community, while the metabolic potentials and ecological importance of fungal community in mangrove sediments have been rarely reported. To this end, the combined approach of third-generation sequencing (PacBio) with Illumina HiSeq technology may overcome the drawback of short reads and offer great benefits to the understanding on the microbial dark matter.

**Fig. 1:**
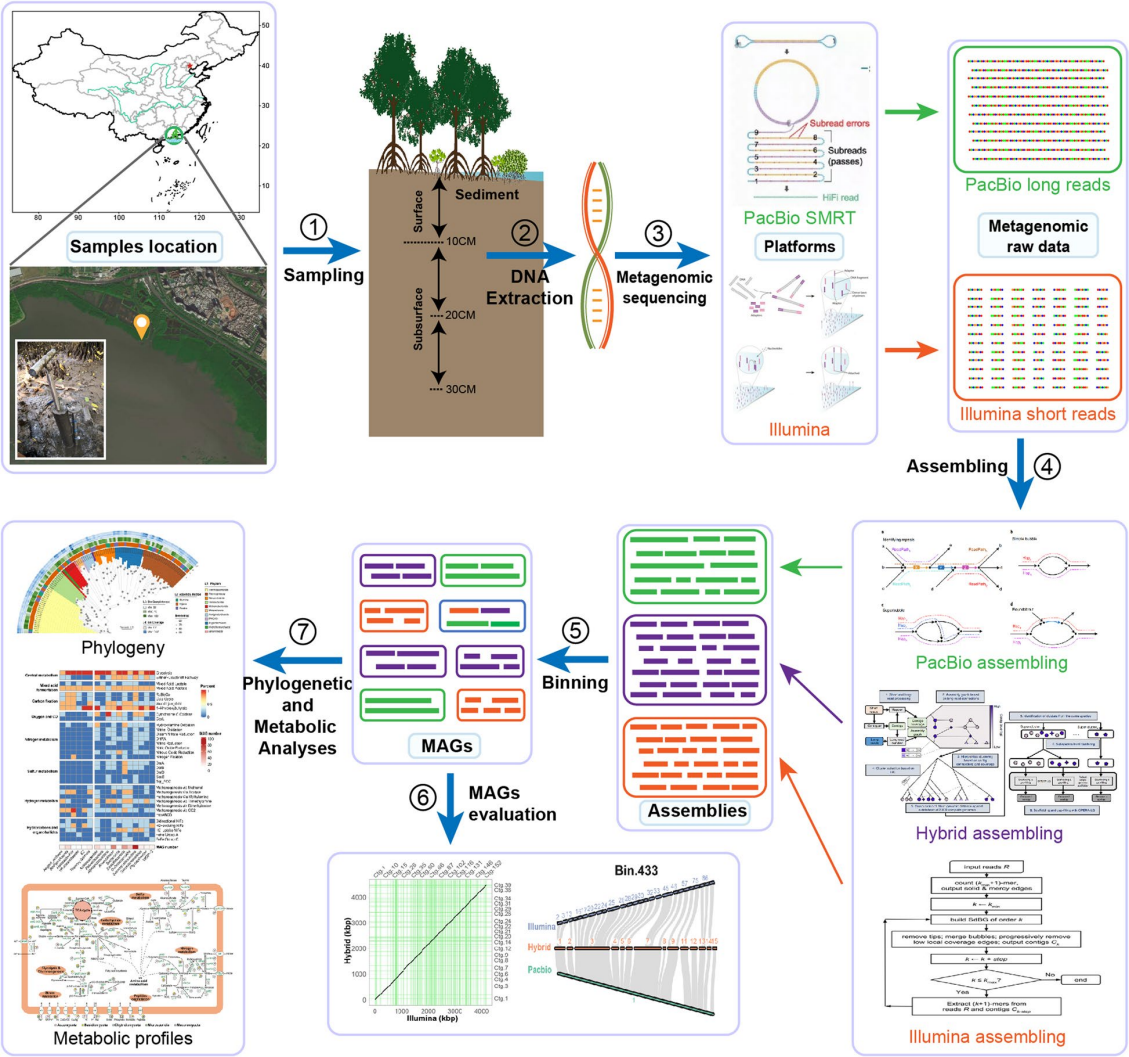
A workflow for the metagenomic study on metabolic profiles of the microbial community in mangrove sediment. The workflow is comprised of seven steps: sample collection, total DNA extraction, metagenomic sequencing, assembling, binning, MAGs evaluation, and phylogenetic and metabolic analyses

Here, we conducted a metagenomic survey based on separate assembly of Illumina short reads, PacBio long reads, and a combination of these two (Hybrid assembly) to investigate the microbial community and metabolic potentials in the mangrove sediment (Fig. 1). Our results show that, comparing to conventional Illumina assembly alone, the supplement of PacBio long reads exhibited great advantages of significantly improving the contig contiguity, reducing the contig number, and yielding new MAGs that represent novel taxa. In addition, Hybrid and PacBio assemblies generated high-quality MAGs as reliable as those produced from Illumina assembly. The study highlights the superiority of Hybrid assembly strategy over Illumina assembly in terms of genome reconstruction and functional characterization of environmental microorganisms and provides us an in-depth understanding of microbial and metabolic profiles in mangrove sediment.

## Materials and methods

### Sample collection and geochemical measurements

Futian Mangrove National Nature Reserve (FT), the only national nature reserve located in an inland urban area in China, was selected. A 30-cm-depth sediment core was collected from the middle site of FT mangrove (22.522294N, 114.014549E) using a steel sampler and separated into three depth layers (0 to 10 cm, 10 to 20 cm, and 20 to 30 cm) in November 2019. Finally, three sediment samples were collected and transferred to laboratory on ice and stored at  -40 °C until analysis. The environmental variables, including salinity, pH, total carbon (TC), total organic carbon (TOC), total nitrogen (TN), ammonium nitrogen (N/NH_4_^+^), nitrate nitrogen (N/NO_3_^−^), total phosphorus (TP), and total sulfur (TS), were determined following the description of Zhang et al. [26].

### Total DNA extraction and metagenomic sequencing

For each sample, DNA was extracted from 10 g of sediments using DNeasy PowerMax Soil kit (Qiagen, Germany) following the manufacturer’s protocol. The quantity and quality of the extracted DNA were examined using a NanoDrop ND-2000c UV–visible-spectrum (UV–Vis) Spectrophotometer (NanoDrop Technologies, USA). For Illumina sequencing, metagenomic data were generated using Illumina HiSeq 2000 instrument at Novogene Bioinformatics Technology Co., Ltd. (Tianjin, China). Approximately 60 Gbp (2 × 150 bp paired-end reads) of raw sequence data were generated for each sample (Dataset S1 Sheet2). For PacBio SMRT sequencing, a 10-kbp length HiFi DNA library was constructed, and metagenomic data was generated using PacBio Sequel II platform in Annoroad Gene Technology Co., Ltd. (Beijing, China). Approximately 180 GB of raw data in bam format was generated for each sample (Dataset S1 Sheet2).

### Microbial diversity analysis

The microbial community were explored using raw Illumina metagenomic reads. To explore prokaryotic community of each sample, 16S rRNA gene fragments were predicated from the raw metagenomic reads using GraftM [43] and then annotated by searching against SILVA database (release 132) [44] using BLASTn [45]. For eukaryotic community, ITS gene fragments were predicated and annotated by searching the raw metagenomic reads against the UNITE and INSDC databases for all eukaryotes (version released on 3 February 2020) [46] using BLASTn. Furthermore, Shannon diversity was calculated using SingleM (https://github.com/wwood/singlem) based on predicated counts of 14 single-copy marker genes [47]. The predicated SingleM counts were rarefied to 100 sequences per maker gene only if > 100 sequences were detected. Diversity was then calculated using Vegan package [48] based on the rarefied SingleM OTU table across each of the 14 marker genes, and the average was taken as the Shannon index for each sample [47].

### Assembly and binning

Metagenomic datasets generated for three layers of sediments were used in a combined assembling. Three assembling strategies, i.e., Illumina-only, PacBio-only, and Hybrid (Illumina and PacBio) assembling, were adopted. For Illumina-only assembly, the raw Illumina reads was dereplicated and trimmed using sickle (https://github.com/najoshi/sickle), and then de novo assembled using MEGAHIT v1.2.9 [49] with the default parameters: -k-list 21, 29, 39, 59, 79, 99, 119, and 141. For PacBio-only and Hybrid assembling, the PacBio ccs reads were extracted and filtered using pbccs v4.02 (https://github.com/PacificBiosciences/ccs) and BAM2fastx tool (https://github.com/pacificbiosciences/bam2fastx/). Subsequently, Hybrid assembling was preformed using OPERA-MS v0.9.0 [13]. In brief, the Illumina-assembled assembly and PacBio ccs reads were supplied as input contig file and long reads file, respectively, with minimap2 as the aligning method for long reads [50]. In PacBio assembling strategy, to make the utmost of the long reads data, method described by Moss et al. [14] and Stewart et al. [15] was adopted. In brief, the PacBio ccs reads were firstly assembled using Canu[51] (parameters: -pacbio-raw and genomeSize = 1g corOutCoverage = 10000 corMhapSensitivity = high corMinCoverage = 0 redMemory = 32 oeaMemory = 32 batMemory = 400, as suggested by developer) and metaFlye [52] (parameters: -pacbio-raw and -meta -i 3) separately. It is reported that Canu and Flye can lead to high accurate assemblies (~ 99.4–99.8% of accuracy) [4, 16]. The two separate assemblies are then polished twice with Illumina clean reads using NextPolish v1.3.1 [53] to reduce errors, which came from the intrinsic error of long-read technologies [4, 14, 15], and subsequently merged using quickmerge v0.30 [54] with parameter -l 500. The merged assembly is the final contigs of PacBio assembling.

Before genome binning, Illumina raw reads were aligned to each of above three assembled contigs using bowtie2 v2.3.5.1 [55] and further processed using SAMtool v1.10 [56]. Each contigs was binned using MetaBAT2 [57], MaxBin 2.0 [58], and VAMB v3.0.1 [59] with default parameters based on sorted bam files. For each assembly method, all bins obtained by these three methods were refined using bin_refinement function in MetaWRAP [60] with parameters -c 30 -x 30. The refined bins for each assembly method were further refined and consolidated into the final bin sets using bin_refinement function with parameters -c 50 -x 10 and double checked by dRep [61]. The completeness, contamination, and strain heterogeneity of binning results were evaluated by using CheckM v1.1.3 [62]. Finally, 562 MAGs with medium quality (completeness ≥ 50%, contamination ≤ 10%) were recovered for following analysis.

To verify the reliability of MAGs from Hybrid assembly and PacBio assembly compared with these from Illumina assembly, twelve final refined MAGs with high quality (completeness ≥ 90, contamination ≤ 10) and at least one draft MAG generated from each of Illumina, PacBio, and Hybrid assemblies, respectively, were selected. Pairwise genome collinearity and gene collinearity of MAGs in each MAG group were analyzed using Mauve v2.4.0 [63] and MCscan pipeline [64] in JCVI utility libraries v0.5.7 (https://github.com/tanghaibao/jcvi), respectively.

### Taxonomy assignments, phylogenetic analysis, and relative abundance of MAGs

Taxonomic assignment of the MAGs was done using the “de novo” workflow of GTDK-Tk toolkit [65] (R202) based on the standardized bacterial and archaeal taxonomy proposed by Parks et al. [66, 67] and Rinke et al. [68] by a combination of three criteria, i.e., placement in the GTDB reference tree, relative evolutionary divergence (RED), and average nucleotide identity (ANI) [65]. Phylogenetic trees were reconstructed for bacterial and archaeal MAGs separately based on concatenation of conserved single-copy genes (120 bacterial marker genes, 122 archaeal marker genes) annotated and aligned by GTDK-Tk toolkit using IQ-TREE2 v2.1.4 [69] with default ModelFinder and parameters -bb 1000 -alrt 1000 and subsequently visualized using iTOL [70]. 16S rRNA genes of each MAGs were predicated and classified by BLASTn searching against SILVA database (release 132) [34]. To estimate the relative abundance of each MAGs at each sediment depth, command “genome” in CoverM v0.6.1 (https://github.com/wwood/CoverM) was executed.

### Functional annotation

To compare overall abundances of metabolic genes at different depths, all quality-controlled Illumina reads were searched against TIGRFAMs [71] and Pfam [72] databases using DIAMOND BLASTx [73] (cutoffs: *e*-value: 1e-10, identity: 70%; best hits reserved) followed the description of Dong et al. [74]. Specific genes encoding hydrogenases, carbohydrate-active enzymes (CAZymes), and dissimilatory sulfite reductases (*dsr*) were further retrieved from quality-controlled Illumina reads through DIAMOND BLASTx queries against comprehensive custom databases [75–78].

For individual MAGs, protein-encoding genes were predicated using Prodigal v2.6.3 [79] (-p meta). Predicated genes were annotated by searching against Kyoto Encyclopedia of Genes and Genomes (KEGG) database [80] using GhostKOALA [81], and the completeness of various metabolic pathways was determined using KEGGDecoder[82] and KEGG-Expander following the default KOALA definitions for metabolic pathways (https://github.com/bjtully/BioData/tree/master/KEGGDecoder). Key genes involved in carbon cycling, nitrogen cycling, sulfur cycling, and other cycling, as well as CAZymes and peptidases, were further identified by HMMER search [83] against default databases, including dbCAN2 [78] and MEROPS [84], using METABOLIC [85].

Specifically, since the MAGs assigned as eukaryotes were low quality (not shown in the results), genes and metabolic functions of eukaryotic community were explored using the assembled eukaryotic contigs. Specifically, the Hybrid assembly was adopted here, due to its largest size and relative less contig number and long contig length. First, eukaryotic contigs were predicted using EukRep v0.6.7 [86]. The potential eukaryotic contigs were subsequently reconfirmed and taxonomic assigned using Kaiju v1.8.2 [87]. Eukaryotic genes were predicated from eukaryotic contigs using MetaEuk [88]. To reconstruct metabolic pathways for specific eukaryotic groups, predicated gene was annotated, and pathway results were summarized using the KEGG server [89, 90].

### Phylogenetic analysis, gene annotation, metabolic pathway reconstruction, and global distribution of candidate new phylum

Genomes of the candidate phylum QNDG01 were retrieved from GTDB database R202 [91] and a new recently published article on marine environments [8]. In total, 12 genomes assigned as QNDG01 were obtained, including four new MAGs in the study. The taxonomy and quality of these genomes were reconfirmed by GTDB-tk [65] and checkM [62], respectively. Phylogenetic analysis of the phylum was processed using IQ-TREE 2 v2.1.4 [69] with default ModelFinder and parameters -bb 1000 -alrt 1000 based on 120 bacterial single-copy marker genes predicated and aligned by GTDB-tk. The adjacent phyla were confirmed by a preliminarily analysis based on GTDB-tk, and their genomes were from GTDB database. rRNA genes of QNDG01 genomes and adjacent phyla were predicated using barrnap v0.9 (https://github.com/tseemann/barrnap), and the tRNA genes were predicated using tRNAscan-SE v2.0.9 [92]. 16S rRNA sequences of QNDG01 genomes and adjacent phyla were aligned using MAFFT [93], and the phylogenetic structure was confirmed by IQ-TREE2. The phylogenetic trees of genomes and 16S rRNA genes were visualized using iTOL. The AAI value shared by any two genomes was calculated using CompareM v0.1.2 with default parameters (https://github.com/dparks1134/CompareM). The ANI value shared by the 16S rRNA genes of any two genomes was determined using pyani v0.2.11 [94].

The protein-encoding genes were predicated by Prodigal v2.6.3 [79] (-p meta). Orthologous gene families were identified using OrthoFinder v2.5.4 [95]. Genes were annotated by searching against KEGG database [80], using GhostKOALA [81], and HMMER [83] searching against dbCAN2 [78] and MEROPS [84], using METABOLIC [85]. Potential metabolic pathways were reconstructed based on above annotations and pathways constructed by KEGG mapper [89, 90].

To determine global distribution of the candidate new phylum, the 16S rRNA gene sequences retrieved were searched against the NCBI nucleotide collection (nt) database and Sequence Read Archive (SRA) database using BASTn [45] and MAPseq [96], respectively. The deposited sequences that shared 83% identity with the query sequences were treated as objects, and their isolation habitats, substrates, location (latitude and longitude), and original publications were recorded. The relative abundance of the 16S rRNA sequences were calculated by dividing the sequence number of the phylum to the total read number of one sample in the MicrobeAtlas website (https://microbeatlas.org).

## Results and discussion

In this study, deep shotgun metagenomic sequencing of mangrove sediment microbiomes was performed with Illumina HiSeq (PE150) and PacBio SMRT sequencing platforms. In total, about 210.9 Gbp of Illumina short reads and 51.5 Gbp of PacBio long reads were obtained (Dataset S1 Sheet2).

### PacBio sequencing method greatly improves the metagenomic assembling and binning

We adopted three assembly strategies, i.e., Illumina assembly, PacBio assembly, and Illumina-PacBio Hybrid assembly, and compared their respective outcome. Size of Hybrid assembly was the largest (8.1 GB), while that of PacBio assembly was the smallest (2.3 GB) (Dataset S2 Sheet1). The number of contigs produced by Illumina assembly was the largest (3,384,302), while that of PacBio assembly was the fewest (48,406). Notably, the longest contig of PacBio assembly (6 219.7 Kbp) was much longer than those of Illumina (357.1 Kbp) and Hybrid (877 Kbp) assemblies. Meanwhile, N50 of PacBio assembly was also the longest (Dataset S2 Sheet1).

We reconstructed 766, 773, and 1451 MAGs from Illumina, PacBio, and Hybrid assemblies, respectively, among which 28, 28, and 47 were high-quality draft genomes (CheckM completeness ≥ 90%, CheckM contamination < 5%) (Fig. S1a and Dataset S2 Sheet2). Subsequently, a total of 562 prokaryotic MAGs with medium quality (completeness ≥ 50%, contamination < 10%) were yielded from all drafts using the bin_refinement function of MetaWRAP [60], and 64 of them were high-quality MAGs (Dataset S2 Sheet2). Among these 562 recovered MAGs, 260 were derived from Illumina assembly (48 Illumina specific), 203 were from PacBio assembly (133 PacBio specific), and 377 were from Hybrid assembly (189 Hybrid specific) (Fig. S1b), indicating that the supplement of PacBio long reads could greatly improve the number of genomes obtained (1.16 times), especially high-quality genomes (1.21 times). To compare the quality of genome reconstruction from different assemblies, we evaluated the contig number, the longest contig, and N50 (Fig. S2). The result showed that the contiguity of PacBio MAGs was substantially increased, with the longest contig of 64.2–6 219.8 kbp (mean = 547.4 kbp) and the largest N50 of 37.2–629.775 kbp (mean = 364.6 kbp), compared to those of Illumina (mean of longest contig: 63.3 kbp, mean of N50: 18.2 kbp) and Hybrid MAGs (mean of longest contig: 129.8 kbp, mean of N50: 47.2 kbp). In addition, there were 1–140 contigs (mean = 31.7) in PacBio MAGs, which were significantly lower than those of Illumina MAGs (3–2 764 contigs, mean = 239.4) and Hybrid MAGs (7–3 636 contigs, mean = 564.5) (Fig. S2).

Next, to evaluate the reliability of MAGs generated from Hybrid and PacBio assemblies, average amino acid identity (AAI) and genome and gene collinearity were analyzed to confirm whether the draft MAGs from three assemblies for the same refined MAGs were exactly the same. Totally, 12 high-quality representative refined MAGs that had at least one high-quality (completeness ≥ 90%, contamination < 5%) draft MAGs from each assembly respectively were selected, and these drafts of the same refined MAGs had high average nucleotide identities (ANI) similarity (> 99%) with each other (Dataset S2 Sheet3). Firstly, the AAI analyses found high similarities among drafts of the same refined MAGs, and most of these values were higher than 99.5% (Fig. S3), which strongly supported the affiliation of these drafts to the same species [97]. Subsequently, after reorder and reverse complement of contigs in draft MAGs, both genome and gene collinearity analyses revealed overall high collinearities (Figs. S4, S5 and S6). The result showed that most collinear blocks in the genomes were found on the forward strand, while only a few collinear blocks were found on the reverse strand in some MAG groups, such as Bin.282, Bin.296, Bin.340, and Bin.429 (Fig. S4). The longest reverse collinearity was found in Bin.296 group, between Illumina-assembled MAG and PacBio-assembled MAG, and the sequence length was about 51 Kbp, comprising only 2.2% of the Illumina-assembled MAG. Collectively, the results of AAI and collinearity analyses demonstrated that the MAGs generated from Hybrid and PacBio assemblies were highly reliable as those from Illumina assembly.

### Quality and diversity of recovered MAGs

Since MAGs generated from Hybrid and PacBio assemblies had considerably high reliability, MAGs from all assemblies refined by MetaWRAP were used for the subsequent analyses. Among 562 prokaryotic MAGs with at least medium quality, 280 were estimated to be > 70% complete, 186 were > 80% complete, 97 were > 90% complete, and 64 were high-quality with completeness ≥ 90% and contamination < 5%. Two MAGs, affiliated with Thermoplasmatota and Zixibacteria, showed 100% completeness and relatively low contamination (1.61% and 1.1%, respectively). Moreover, four MAGs had only one contig, and three of them were not contaminated (0% contamination), and one, i.e., Bin.433, was almost complete (95.7% completeness) (Dataset S2 Sheet4). According to the assignment of GTDB-Tk, only 12 MAGs reconstructed in this work had been previously reported in other studies, and the remaining 550 MAGs were newly reconstructed (Dataset S2 Sheet4).

**Fig. 2:**
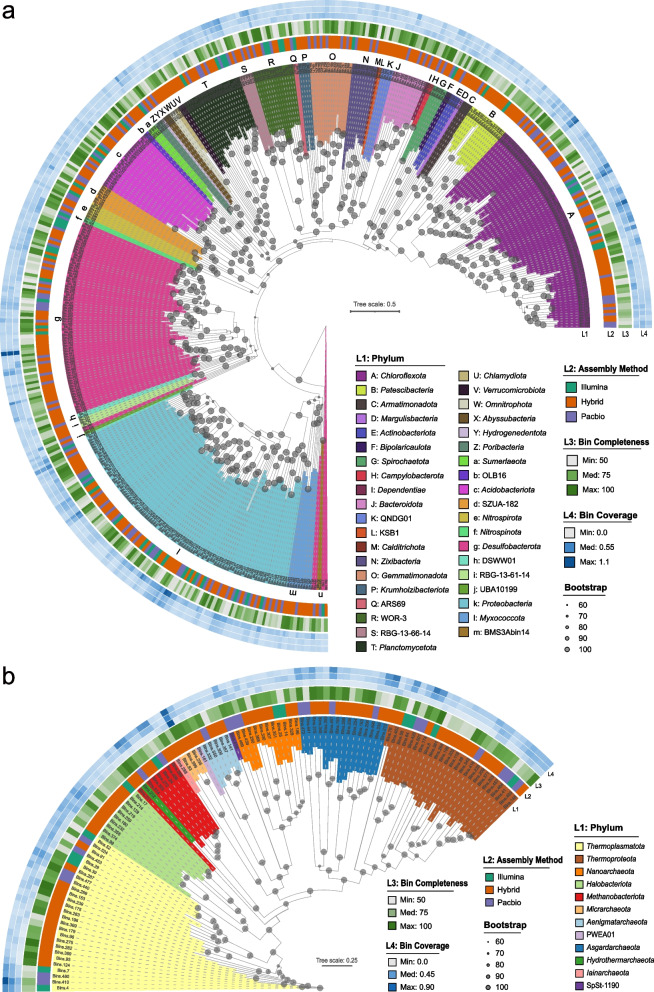
Phylogenetic tree of retrieved MAGs based on single-copy genes. **a** Phylogenetic tree of bacterial MAGs based on 120 bacterial single-copy genes. **b** Phylogenetic tree of archaeal MAGs based on 122 archaeal single-copy genes. The clade labels are colored according to bacterial and archaeal phylum as outer layer L1. Outer layer L2 to L4 of the trees represent the assembly method, completeness, and coverage depth of each MAG, respectively. Bootstrap values are labeled with gray solid circles

Since only 240 MAGs contained fragments of the 16S rRNA gene (> 300 bp), phylogenetic analysis based on concatenated conserved genes obtained by GTDB-Tk [65] was performed to determine the taxonomic position of 562 refined MAGs (Fig. 2 and Dataset S2 Sheet4). Recovered MAGs spanned 39 bacterial phyla (455 MAGs) and 12 archaeal phyla (107 MAGs), most of which were poorly characterized without cultured representatives. Bacterial MAGs were mostly represented by phyla Proteobacteria (95 MAGs), Chloroflexota (80 MAGs), and Desulfobacterota (78 MAGs) and by class Gammaproteobacteria (84 MAGs), *Anaerolineae* (53 MAGs), and Desulfobacteria (45 MAGs), while archaeal MAGs were mostly represented by phyla Euryarchaeota (27 MAGs), Bathyarchaeota (24 MAGs), and Asgard archaea (15 MAGs) (Dataset S2 Sheet4). The composition of recovered MAGs is consistent with the prokaryotic community composition detected by the 16S rRNA gene from Illumina raw data (Fig. S7a, Fig. S8a, and Dataset S1).

The potential eukaryotic MAGs were evaluated using BUSCO [98], following the strategy adopted by Alexander et al. [99] and Delmont et al. [100]. However, these MAGs were with low completeness (not shown in the results) for two possible reasons. First, the assembly of eukaryotic genomes from complex community remains one of the major computational challenges because of the diploid or polyploid nature and the existing of long repetition [86]. Furthermore, the micro-eukaryotes are of a relatively low proportion of microbial community in mangrove sediment (as low as 9% of the retrieved marker genes from Illumina raw data, Fig. S7, Fig. S8, and Dataset S1). Therefore, genes and metabolic potentials of eukaryotic community were explored using the assembled contigs from the Hybrid assembly because of its largest size, relatively low contig number, and long contig length. By EukRep predection [86] and Kaiju annotation [87], 33 800 contigs, *ca.* 163 Mbp, were classified as eukaryotic, of which the longest length was about 199 Kbp and the mean length was ca. 5 Kbp.

### Metabolic details of microbial community

So far, detailed investigation of the metabolic function of fungal community in mangrove sediment is lacking, since most previous studies focused on prokaryotes and a few research explored the fungal community solely based on ITS metabarcoding [26, 101]. Using 562 median- and high-quality prokaryotic MAGs and 9 710 assembled fungal contigs, we preliminarily explored carbon-, nitrogen-, and sulfur-related metabolic potentials of microbial community in mangrove sediment.

#### Carbon metabolism

##### Versatile catabolic capabilities of prokaryotes for complex carbon substrates

Metabolic reconstructions of 562 MAGs revealed versatile catabolic capabilities for assimilating carbohydrates, peptides, and short-chain fatty acids (Fig. S10 and Dataset S2). Generally, the analysis revealed numerous cellulose, hemicellulose, and other polysaccharide degradation genes in most recovered phyla (Fig. S10, Dataset S2 Sheet7), suggesting an important role of mangrove sediment microbiota in the degradation of complex organic carbon compounds [102, 103]. Specifically, some MAGs belonging to the phyla Armatimonadota, Hydrogenedentota, OLB16, Planctomycetota, Poribacteria, and Verrucomicrobiota contained the most abundant glycoside hydrolase (GH) genes (total genes > 70, gene density > 10 genes/Mbp). Besides, Hydrothermarchaeota, Acidobacteriota, Bacteroidota, Chloroflexota, QNDG01, RGB-13–66-14, Spirochaetota, and Sumeriaeota had relatively high number of GH genes (total genes > 40, gene density > 6.5 genes/Mbp) (Fig. S10, Dataset S2 Sheet7). In addition, we found a number of genes that participated in the degradation of chitin, a long-chain N-acetylglucosamine polymer compound that comprises arthropod exoskeletons, fungal, and algal cell wells [103]. Endo-acting chitinase genes, which randomly cleave glycosidic linkages in chitin and chitodextrins in a non-processive mode, were widespread in Acidobacteriota, Bacteroidota, Chloroflexota, and Proteobacteria MAGs. N-acetyl-glucosaminidase genes were found in a number of Acidobacteriota, Bacteroidota, Chloroflexota, Desulfobacterota, Planctomycetota, and Proteobacteria MAGs (Dataset S2 Sheet7).

In sediment, proteins are one of the most important bioavailable carbon and nitrogen sources [102, 103]. According to the annotation against MEROPS database (Release 12.4) [84], 561 of 562 recovered MAGs encoded putative peptidase genes, and the largest number of genes was identified in bacterial phyla Acidobacteriota, Bacteroidota, Chloroflexota, Desulfobacterota, Planctomycetota, and Proteobacteria and candidate phyla QNDG01 (> 50 putative peptidase genes, Dataset S2 Sheet8). In contrast, MAGs of archaeal phyla tended to have less peptidase genes, such as Woesearchaeota, Bathyarchaeota, Euryarchaeota, and Aenigmatarchaeota (Dataset S2 Sheet8). This result indicated that mangrove bacteria might be fundamentally involved in the protein degradation in mangrove sediment.

Fatty acids play essential roles in membrane structure, architecture, homeostasis, and transport and also constitute important sources of metabolic energy [104]. Acetyl-coenzyme A (CoA) generated from fatty acids and organic acids via β-oxidation pathway is metabolized to obtain energy and precursors for cellular biosynthesis [104–106]. The β-oxidation pathway could be detected in 70% of recovered MAGs, which belonged to bacterial phyla Desulfobacterota, Spirochaetota, Proteobacteria, Abyssubacteria, Chloroflexota, and Myxococcota and candidate phyla DSWW01 and RGB-13–66-14. In particular, 22 of 45 MAGs with more than 10 β-oxidation genes were Desulfobacterota (Dataset S2 Sheet6), suggesting that Desulfobacterota possibly played important role in the degradation of fatty acids in anaerobic sediment conditions [107]. The distribution of those MAGs with most β-oxidation genes along sediment depth was not consistent, half of which (23 MAGs) were more abundant in surface sediment, while other MAGs (22 MAGs) were more abundant in subsurface sediment (Dataset S2 Sheet4 and Sheet6).

##### Broad metabolic potentials of fungi in organic carbon

**Fig. 3:**
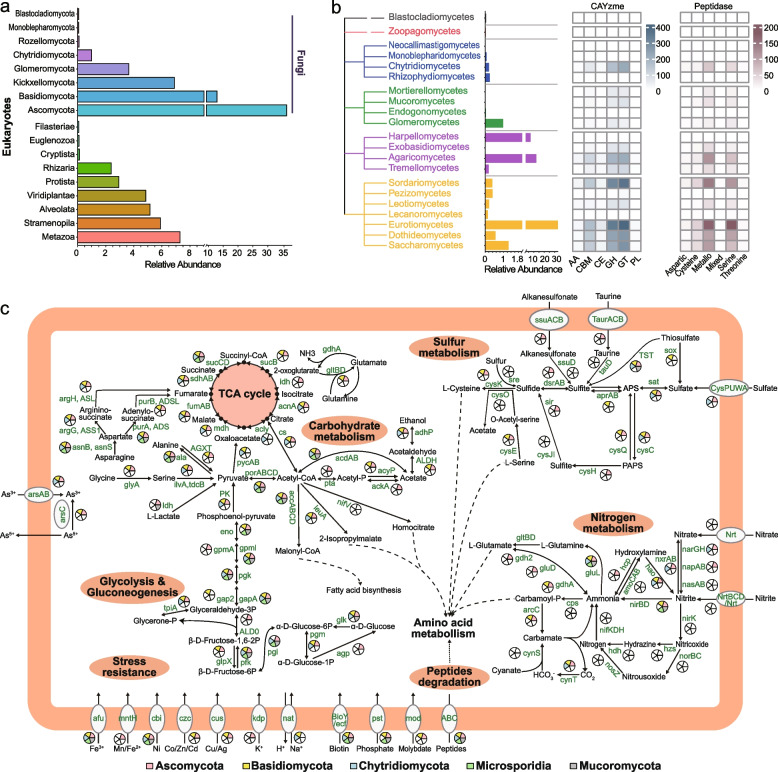
Composition and metabolic functions of the microeukaryotic community in mangrove sediment. **a** The relative abundance of different eukaryotic groups within total eukaryotes. **b** The profiles of carbohydrate-active enzymes (CAZymes) and peptidase families identified in major fungal groups in mangrove sediment. The number of detected genes is denoted by color shades. Abbreviations: GH, glycosidases or glycosyl hydrolases; PL, polysaccharide lyases; CE, carbohydrate esterases; GT, glycosyltransferases; AA, auxiliary activities; CBM, carbohydrate-binding modules. **c** Metabolic potentials of carbon, nitrogen, and sulfur metabolism in five dominant fungal groups. The presence of genes within the metabolic pathways of each phylum is denoted by the area in pie chart with colors indicating corresponding phylum. Gene symbols and metabolites are labeled with the KEGG designation (details in Dataset S3 Sheet 1)

In total, 6509 CAZymes (249 families) and 2486 peptidases (109 families) were detected (Fig. 3b, Dataset S3 Sheet2 and Sheet3) in fungal contigs. Observed CAZymes comprised a wide range of enzyme families, including 105 GHs (mostly GH0, GH13, and GH43), 59 GTs (glycosyltransferases, mostly GT4, GT1, and GT0), 42 CBMs (carbohydrate-binding modules, mostly CBM50, CBM13, and CBM48), 18 PLs (polysaccharide lyases, mostly PL0, PL12, and PL9), 13 CEs (carbohydrate esterases, mostly CE11, CE4, and CE15), and 12 AAs (auxiliary activities, mostly AA1, AA5, and AA4). The majority of CAZymes falling into diverse enzyme families were distributed in several fungal classes, for instance Eurotiomycetes (996 CAZymes of 169 families), Sordariomycetes (872 CAZymes of 141 families), Dothideomycetes (679 CAZymes of 138 families), Saccharomycetes (674 CAZymes of 125 families), Agaricomycetes (565 CAZymes of 133 families), and Chytridiomycetes (462 CAZymes of 104 families). Particularly, key CAZyme genes encoding putative xylanase (GH10) were found in Dothideomycetes, Eurotiomycetes, Exobasidiomycetes, Leotiomycetes, Schizosaccharomycetes, and Sordariomycetes. Putative cellulase-encoding sequences (GH5) were found in several classes such as Eurotiomycetes, Dothideomycetes, Sordariomycetes, Saccharomycetes, Leotiomycetes, Mortierellomycetes, and Ustilaginomycetes (Dataset S3 Sheet2), indicating their possible degradation capability of cellulose.

Eight peptidase groups were found in the fungal contigs, i.e., aspartic (A), cysteine (C), inhibitors (I), metallo (M), asparagine lyase (N), mixed (P), serine (S), and threonine (T) peptidases (Fig. 3b and Dataset S3 Sheet3). Among them, metallopeptidases were the most abundant (1072 genes of 41 families), followed by serine (832 genes of 24 families), cysteine (312 genes of 21 families), aspartic (83 genes of six families), asparagine (14 genes of three families), and mixed catalytic peptidases (12 genes of one families). Peptidase genes were abundant in Eurotiomycetes (429 genes of 80 families), Sordariomycetes (333 genes of 66 families), Dothideomycetes (248 genes of 62 families), Saccharomycetes (243 genes of 62 families), Agaricomycetes (226 genes of 58 families), Chytridiomycetes (136 genes of 47 families), and Tremellomycetes (81 genes of 38 families) (Dataset S3 Sheet3). The above observations indicate that fungi in mangrove sediments have broad metabolic potentials in organic carbon cycling and degradation of various carbohydrate and peptide substrates.

##### Widespread capacities of carbon fixation in prokaryotes

**Fig. 4:**
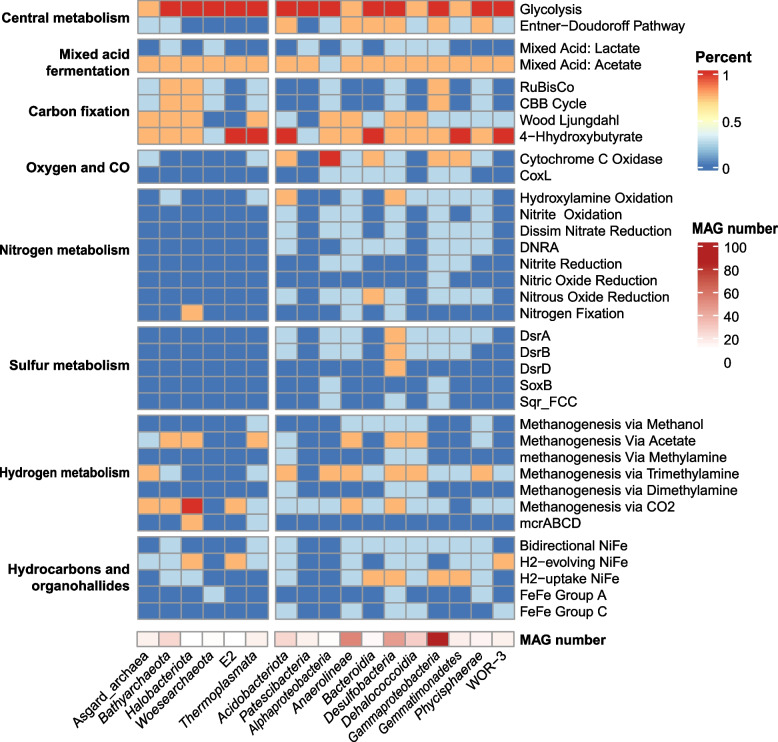
Functional profiles of the main microbial groups in mangrove sediment. The occurrence percentages of core metabolic genes or pathways are calculated by dividing the number of MAGs with genes or pathways present by the MAG number of each phylogenetic cluster. Complete lists of metabolic genes or pathways can be found in Dataset S2 Sheet5. Detailed gene lists for each pathway indicated can be found at https://github.com/bjtully/BioData/blob/master/KEGGDecoder/KOALA_definitions.txt. The left part of heatmap belongs to archaeal MAGs, and the right is bacterial MAGs. The bottom raw represents the MAG number of each phylogenetic cluster

Unlike photosynthetic organisms, chemoautotrophic microbes acquire energy to synthesize organic compounds by oxidizing inorganic compounds, such as ammonia (NH_3_), carbon monoxide (CO), hydrogen, hydrogen sulfide (H_2_S), and metals [108, 109]. Overall, we observed three distinct carbon fixation pathways in recovered MAGs, including Calvin-Benson-Bassham (CBB) cycle (118 MAGs), reverse tricarboxylic acid (rTCA) cycle (two MAGs), and Wood-Ljungdahl (WL) pathway (272 MAGs) (Fig. 4, Fig. S11, and Dataset S2 Sheet5). Specifically, CBB cycle, the predominant atmospheric CO_2_ fixation pathway that widely distributed in most autotrophic organisms including plants, algae, cyanobacteria, and some autotrophic bacteria [110], was mainly found in Proteobacteria, Bathyarchaeota, Euryarchaeota, Halobacteriota, Asgard archaea, and Methanobacteriota, while WL pathway was mainly in Chloroflexota, Desulfobacterota, Planctomycetota, Acidobacteriota, Bathyarchaeota, Euryarchaeota, Asgard archaea, Halobacteriota, and Nitrospirota. Interestingly, several archaeal MAGs of Bathyarchaeota, Halobacteriota, Asgard archaea, Euryarchaeota, and Methanobacteriota have both CBB cycle and WL pathway (Fig. 4, Fig. S10, and Dataset S2 Sheet5 and Sheet6). However, key genes of these autotrophic pathways were not found in fungal contigs. Collectively, the results suggest that prokaryotes in mangrove sediment have various pathways for carbon fixation [38].

#### Nitrogen metabolism

Metabolic pathway reconstruction from recovered MAGs revealed that the mangrove prokaryotic community possessed the complete nitrate reduction (both dissimilarity and assimilatory pathways), denitrification, and nitrogen fixation pathways. A partial nitrification pathway was found, but genes involved in anammox were lacking (Fig. 4, Fig. S11, and Dataset S2 Sheet5 and Sheet6). In anaerobic respiration, dissimilatory nitrate reduction to ammonia (DNRA), denitrification, and sulfate reduction were three important energy-producing pathways, with nitrate/nitrite or sulfate being the electron acceptors [111–113]. In this study, key genes for DNRA, including *narGH*/*napAB* and *nirBD/nrfAH*, and denitrification pathways, including *nirKS*, *norBC*, and *nosZ*, were found in a total of 228 MAGs (40.6%), most of which belonged to bacterial phyla Proteobacteria (81 MAGs), Desulfobacterota (38 MAGs), and Chloroflexota (27 MAGs) (Fig. 4, Fig. S11, and Dataset S2 Sheet5 and Sheet6), indicating that these N-related pathways might represent essential energy sources for particular bacterial groups. On the other hand, nitrification, a two-step process that aerobically oxidizes ammonia to nitrate with hydroxylamine and nitrite as intermediate products [114, 115], was partially observed in 170 MAGs. In addition, 27 MAGs of Chloroflexota, Desulfobacterota, Halobacteriota, Methanobacteriota, and Myxococcota possess genes encoding nitrogenases (Dataset S2 Sheet5 and Sheet6), which catalyze the biological reduction of dinitrogen to ammonia [116]. These observations indicate the great ecological roles of prokaryotes in nitrogen cycling in mangrove sediment.

Similar to the prokaryotic community, the fungal community in mangrove sediment was found to possess the complete dissimilarity nitrate reduction pathway and a partial nitrification pathway (Fig. 3c, Dataset S3 Sheet1). The key enzymes for dissimilatory nitrate reduction were found in several fungal groups, while those for assimilatory nitrate reduction were absent in all fungal groups, indicating that the former pathway might be an important function for the mangrove fungi. Furthermore, nitrification pathway was partially found, with *amoCAB* genes missing, indicating the lack of ability of converting ammonia to hydroxylamine [115]. Downstream, the presence of *hao* and *nxrAB* genes indicated the potential capability of catalyzing hydroxylamine to nitrite and nitrate step by step [115] (Fig. 3c). Despite similar N-related metabolic potentials observed, the mangrove fungal community likely played a distinct role compared to the prokaryotic community. Although studies have revealed that fungi may play and activate role in denitrification, and several isolates of *Aspergillus*, *Fusarium*, *Penicillium*, and *Tritirachium* are capable of anaerobic denitrification in anoxic sediment habitats [117, 118], the fungal community in current study appeared to lack the key enzymes involved in nitrogen denitrification (*nirKS*, *norBC*, and *nosZ*) and anammox (*hzs* and *hdh*), possibly due to the insufficient fungal metagenomic data (Fig. 3c). Altogether, these observations suggest that the fungal community may have important ecological significance in the nitrogen cycling in mangrove sediment.

#### Sulfur metabolism

Sulfate reduction is one of the main anaerobic respiratory pathways that many anaerobic microbes depend on [113]. The prokaryotic community in mangrove sediment harbored the complete pathways for both assimilatory and dissimilatory sulfate reduction (Dataset S2 Sheet5 and Sheet6). Some prokaryotic members, represented by 35 MAGs of Proteobacteria, Chloroflexota, and Desulfobacterota, could uptake extracellular sulfate from surrounding environments by ABC transporters (encoded by *cysUWA* genes). Sulfate within cells were then converted to sulfide in assimilatory and dissimilatory ways. The key genes for assimilatory sulfate reduction, including *cysC*, *cysH*, and *cysJ*, were detected in 146 MAGs, most of which belonged to bacterial phyla Proteobacteria (33 MAGs), Desulfobacterota (15 MAGs), and Planctomycetota (10 MAGs) and archaeal phyla Euryarchaeota (20 MAGs), Asgard archaea (11 MAGs), and Halobacteriota (9 MAGs). The key genes for dissimilatory sulfate reduction, including *aprAB* and *dsrAB*, were found in 150 MAGs, mainly belonging to bacterial phyla Desulfobacterota (50 MAGs), Proteobacteria (47 MAGs), and Acidobacteriota (10 MAGs). In contrast, relevant key genes were not detected in archaeal MAGs. The gene for sulfate adenylyltransferase (*sat*) that catalyzes upstream reduction of sulfate to APS (adenylyl sulfate) was observed in 229 MAGs, most of which were bacteria (207 MAGs), mainly Proteobacteria (45 MAGs), Desulfobacterota (44 MAGs), Chloroflexota (37 MAGs), and Acidobacteriota (18 MAGs), and a small proportion were archaea (22 MAGs) (Dataset S2 Sheet5 and Sheet6). The wide possession of complete sulfate reduction enzymes indicated that the sulfate reduction might be an important energy-producing pathway for microbes in mangrove sediment [113]. In addition, 57 MAGs, mainly Proteobacteria (54 MAGs), possess *sox* genes, indicating their potential in thiosulfate/sulfide oxidization (Dataset S2 Sheet5 and Sheet6). In summary, these results highlight the predominant role of bacteria in sulfur cycling in mangrove sediment.

Similar to the bacteria and archaea, data from assembled fungal contigs suggest that fungi in mangrove sediment are involved in the natural sulfur cycling, which has not been reported in mangrove. The sediment fungi contained key genes for both assimilatory and dissimilatory sulfate reduction and sulfide oxidation (Fig. 3c). A large number of genes that catalyze the conversion of sulfate to sulfide such as *sat*, *cysC*, *cysH*, and *sir* were detected. The *sat* gene was widely detected in Ascomycota (7 genes in 5 classes), Basidiomycota (8 genes in 2 classes), and Chytridiomycota (1 gene in 1 class) (Dataset S3 Sheet1). The presence of key genes for sulfate reduction pathways indicated the potential of mangrove fungi in using sulfate reduction for energy in mangrove sediment. For oxidation of sulfide to sulfate, *dsrA*/*B* genes were detected in Eurotiomycetes and Saccharomycetes in Ascomycota and Mortierellomycetes in Mucoromycota, *aprA*/*B* genes were detected in Agaricomycetes and Exobasidiomycetes, and *sat* gene was detected in eight classes in Ascomycota, Basidiomycota, and Chytridiomycota (Fig. 3c). Overall, the detection of the pivotal metabolic genes involved in the sulfur metabolism suggested the potential role of fungal community in sulfur and energy cycling in the mangrove sediment [113].

#### Hydrogen metabolism

H_2_ metabolism is proposed to be the most ancient and diverse energy conservation mechanism [75]. There are three types of hydrogenases, *NiFe*, *FeFe*, and *Fe* hydrogenases, distinguished by their metal composition [75, 119]. In total, 359 out of 562 recovered MAGs encoded hydrogenases in this study, mostly of which were *NiFe* hydrogenases (341 MAGs), and a few were *FeFe* (54 MAGs). The *NiFe* hydrogenases are classified into four groups, namely group 1 (respiratory H2-uptake hydrogenases), group 2 (alternative and sensory uptake hydrogenases), group 3 (cofactor-coupled bidirectional hydrogenases), and group 4 (respiratory H2-evolving hydrogenases). On one hand, the majority of *NiFe*-encoding MAGs (192 MAGs) encoded bidirectional *NiFe* (group 3), which were predicted to participate in both the fermentative production of H_2_ and sulfate reduction with hydrogen being the electron donor [75, 103]. These genes were distributed in several bacterial phyla, including Chloroflexota (38 MAGs), Desulfobacterota (20 MAGs), Proteobacteria (20 MAGs), Acidobacteriota (12 MAGs), and Planctomycetota (10 MAGs), and two archaeal phyla Bathyarchaeota (21 MAGs) and Euryarchaeota (15 MAGs) (Dataset S2 Sheet6). Meanwhile, genes encoding H_2_-uptake *NiFe* (group1, in 160 MAGs) that involved in H_2_ consumption were pervasive in bacteria (159 MAGs), mostly Proteobacteria (46 MAGs), Desulfobacterota (38 MAGs), and Chloroflexota (23 MAGs), indicating the metabolic potentials of organic carbon degradation and fermentation [75]. On the other hand, genes encoding the primary H_2_-producing *NiFe* (group 4) [75, 120] were identified in 101 MAGs affiliated with 16 bacterial and seven archaeal phyla. Several MAGs in Desulfobacterota, Chloroflexota, Armatimonadota, and Bathyarchaeota encoded both H_2_-uptake and H_2_-evolution *NiFe* genes, suggesting the possible genomic capabilities of both H_2_ production and consumption of these taxa (Dataset S2 Sheet6). Overall, the prevalent distribution of hydrogenases among fermenting and respiring prokaryotes suggests that H_2_ may be a highly dynamic electron carrier produced and consumed by a wide range of microbes in mangrove sediments, as revealed in other anoxic environments [103, 121].

### New candidate phylum *Candidatus* Cosmopoliota

#### The propose new bacterial phylum *Ca.* Cosmopoliota

**Fig. 5:**
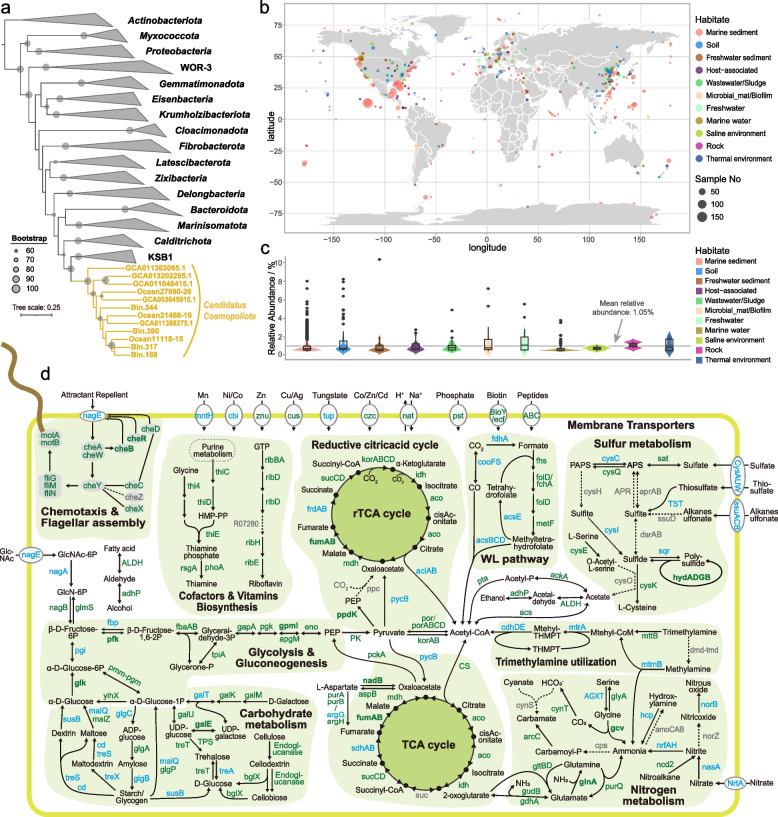
Phylogeny, distribution, and metabolic pathways of the new phylum *Candidatus* Cosmopoliota. **a** Phylogenetic relationship of *Ca.* Cosmopoliota and other bacterial phyla based on 120 bacterial single-copy genes. Taxonomic information of the reference genomes is obtained from the GTDB r202 database. The MAGs in *Ca.* Cosmopoliota are labeled in orange color with bold font. The nodes with bootstrap > 60% are labeled with gray solid circles. **b** Reconstruction of the key metabolic pathways of *Ca.* Cosmopoliota. Solid arrows indicate genes identified in at least one MAG, and dashed arrows indicate genes absent in all MAGs. Genes in gray color are absent in all MAGs, those in blue color are identified in less than half of the MAGs, those in green color are present in more than half of the MAGs, and those in green color and bold font are present in all MAGs. Details of genes and the gene distribution are in Dataset S4 Sheet2. WL pathway, Wood-Ljungdahl pathway. **c** Global distribution of *Ca.* Cosmopoliota. The distribution is investigated based on 16S rRNA genes. Detail information is provided in Dataset S4 Sheet6 and Sheet7

In the study, four new bacterial MAGs were assigned to QNDG01 lineage by GTDB-Tk assignment [65] (Fig. 2). Subsequently, we downloaded eight genomes belonging to QNDG01, including five genomes from GTDB database r202 [91], and three MAGs from an analysis on marine environments [8]. Phylogenetic analysis using 120 bacterial single-copy maker genes and 16S rRNA gene sequences revealed an almost consistent topology of the trees, where the QNDG01 lineage was the most closely clustered with KSB1 and Calditrichota clades (Fig. 5a and S12).

Comparison of 16S rRNA genes is a common approach to assess the taxonomy of newly constructed genomes. In current study, four 16S rRNA genes with relatively long length (808–1506bp) were retrieved from QNDG01 genomes, with one from the newly obtained MAGs (Dataset S4 Sheet1). The 16S rRNA gene sequences from QNDG01 genomes shared similarities from 84.0 to 91.7% with each other, while the similarities were lower than 83.1% between QNDG01 genomes and adjacent phyla (Fig. S14). We further analyzed ANI and AAI shared by genomes of QNDG01 and related phyla. The result showed a relatively lower genomic ANI between QNDG01 genomes and adjacent phyla KSB1 (66.3–69.1%, mean = 67.8%) and *Calditrichota* (66.5–69.6%, mean = 68.0%). Furthermore, the genomes within QNDG01 lineage shared high AAI values with each other (> 53%), which were significantly higher (*P* < 0.001) than that shared by QNDG01 genomes with adjacent KSB1 (47.1–51.5%, mean = 49.7%) and *Calditrichota* lineages (46.4–49.7%, mean = 48.1%) (Fig. S13). Based on the combined results of ANI, phylogenetic and phylogenomic analyses, we propose the QNDG01 lineage as a new candidate phylum [2, 97, 122].

#### Environmental distribution

To investigate the distribution and abundance of the newly proposed candidate phylum, the 16S rRNA gene sequences retrieved from the genomes were used to search in GenBank and Sequence Read Archive (SRA) data in NCBI by BLASTn [36, 123]. In total, we found 1607 datasets with geographic information that contained sequences sharing at least 83% sequence identity with the 16S rRNA genes of the proposed phylum*.* These included 1450 SRA datasets and 157 sequences from GenBank (Dataset S4 Sheet6 and Sheet7). The result showed that members of the new phylum were ubiquitously distributed in all types of ecosystems on Earth (Fig. 5b). We roughly classified 1607 sites into 11 kinds of habitat and found that this new phylum was the most frequently detected in marine sediment (70.9% of all sites, Dataset S4 Sheet6-Sheet7), where the dissolved oxygen content and turnover rate of nutrients are generally low [123, 124]. Its highest relative abundance (10.3%) was found in a freshwater lake sediment in Australia (SRA: ERS411372, BioProject: PRJEB5714; Dataset S4 Sheet7, Fig. 5c). The distribution unveils that species of the proposed phylum possibly prefer oxygen-limited niches, such as anoxic aquatic sediment [123], but can be hypoxic tolerant as members were also detected in aerobic environments such as water and soil.

Following the guidance for naming uncultivated bacteria [97, 125, 126], we proposed the name *Candidatus* Cosmopoliota for this phylum, according to their cosmopolitan distribution. The taxonomic description is provided in Supplementary Materials.

#### Metabolic potential of *Ca.* Cosmopoliota

The data showed that members of *Ca.* Cosmopoliota may utilize various sugar compounds, peptides, various amino acids, and short-chain fatty acids as carbon and energy resources (Fig. 5d). In these 12 genomes of Cosmopoliota, a number of genes for degradation of various sugar compounds are identified, including genes for the hydrolysis of glucose (*glk* in 12 genomes), galactose (*galK* in 11 genomes), fructose (*scrK* in 10 genomes), starch/glycogen/maltose (*glgP*/*malQ* in 6 genomes), and some plant- or animal-derived polysaccharides (GH28, GH113, and GH114) (Dataset S4 Sheet2 and Sheet3). In addition, genomes of *Ca.* Cosmopoliota harbor a number of genes for peptidase and genes encoding enzymes that convert amino acid to pyruvate, such as glutamate dehydrogenase (*gdhA* in 6 genomes), threonine dehydratase (*tdcB* in 9 genomes), adenylosuccinate synthase (*purAB* in 12 genomes), L-aspartate oxidase (*nadB* in 12 genomes), and aspartate aminotransferase (*aspB* in 8 genomes) (Dataset S4 Sheet2 and Sheet4). These bacteria are capable of utilizing short-chain fatty acids, such as propionate, as carbon and energy sources, because they have the complete gene sets for the conversion of propionate to succinyl-CoA, including *acs* (8 genomes), *ackA* (11 genomes), *pta* (10 genomes), *pccB* (6 genomes), *epi* (6 genomes), and *mcmA* (8 genomes) (Dataset S4 Sheet2).

Furthermore, members of the new phylum have the complete glycolysis pathway and may produce acetate, lactate, and alcohol as fermentation products (Fig. 5d). Whereafter, acetyl-CoA may be produced by catalysis of pyruvate ferredoxin oxidoreductase (*por* in 12 genomes) and 2-oxoglutarate ferredoxin oxidoreductase (*kor* in 10 genomes) (Fig. 5d, Dataset S4 Sheet2). Meanwhile, genes encoding acetyl-CoA synthetase (*acs* in 8 genomes), acetate kinase (*ackA* in 11 genomes), phosphate acetyltransferase (*pta* in 10 genomes), and lactate dehydrogenase (*ldh* in 10 genomes) are observed, indicating that acetate and lactate are possible fermentation products (Fig. 5d, Dataset S4 Sheet2). Furthermore, the presence of genes encoding aldehyde dehydrogenase (ALDH) and alcohol dehydrogenase (*adhP* in 7 genomes) indicates the capability of alcohol fermentation (Fig. 5d).

Interestingly, 11 genomes contain genes for *NiFe* group 4a–g, suggesting that these bacteria may use H^+^ as the respiratory electron acceptor and produce and release H_2_ (Dataset S4 Sheet5) [75]. Meanwhile, eight of the 11 H_2_-evolving genomes also have genes for *NiFe* group 3c, which can direct electrons from H_2_ to heterodisulfide and ferredoxin [75, 127]. Thereby, these bacteria may consume hydrogen gas during energy production and conversion. Besides, genes for cytochrome c oxidases that drive oxidative phosphorylation were absence in most genomes (Dataset S4 Sheet2), indicating that anaerobic fermentation may be an important energy-producing process in *Ca.* Cosmopoliota.

In the bacteria, both assimilatory and dissimilatory nitrate reductions are incomplete, and only two genes involved are observed, i.e., assimilatory nitrate reductase (*nasA* in 1 genomes) and nitrite reductase (*nrfAH* in 2 genomes). Instead, some members of the new phylum can convert trimethylamine and methylamine to acetyl-CoA and ammonia, respectively. In addition, most members have the ability to convert nitroalkane to nitrite by nitronate monooxygenase (*ncd2* in 7 genomes) (Fig. 5d, Dataset S4 Sheet2). For sulfur cycling, species in this phylum possibly catalyze the transformation of some inorganic and organic sulfur compounds because of the presence of genes *sseA* for thiosulfate (4 genomes), *hydADGB* for polysulfide (12 genomes), and *dcyD* for cysteine (3 genomes). Furthermore, the phylum harbors the almost complete assimilatory sulfate reduction pathway, except the gene for phosphoadenosine phosphosulfate reductase (*cysH*), which catalyzes 3′-phosphoadenylyl sulfate (PAPS) to sulfite (Fig. 5d, Dataset S4 Sheet2).

Notably, both the WL pathway and the rTCA cycle are detected in phylum *Ca.* Cosmopoliota. The WL pathway is an important component of the metabolic machinery, playing crucial roles in microbial energy conservation and carbon assimilation in diverse anaerobic prokaryotes [123, 128]. The pathway fixes two carbon dioxide molecules to acetyl-CoA using the methyl branch or the carbonyl branch and can operate in the reductive (acetyl-CoA formation from CO_2_) and oxidative (acetate degradation to two C1 compounds) directions [129, 130]. In the study, the WL pathway detected in *Ca*. Cosmopoliota is predicted to operate in the reductive direction. In short, in the methyl branch, CO_2_ is progressively reduced to methyl by formate dehydrogenase (*fdh* in 5 genomes) and eventually converted to methyltetrahydrofolate (methyl-THF) by enzymes formate-tetrahydrofolate ligase (*fhs* in 10 genomes), methylenetetrahydrofolate dehydrogenase (*folD* in 11 genomes), and methylenetetrahydrofolate reductase (*metF* in 7 genomes) step by step. Meanwhile, in the carbonyl branch, a CO_2_ molecule is reduced to CO (carbonyl moiety) by anaerobic carbon-monoxide dehydrogenase (*coo* in 2 genomes). Under the subsequent catalysis of *acsBCD* (2 genomes), the CO molecule is combined with the methyl group in methyl-THF from the methyl branch and CoA to form acetyl-CoA (Fig. 5d, Dataset S4 Sheet2) [128, 130]. The rTCA cycle is essentially the oxidative TCA cycle running in reverse, leading to the fixation of two CO_2_ molecules to one molecule of acetyl-CoA [131]. In this study, we found that *Ca.* Cosmopoliota harbors a complete set of genes for the rTCA cycle, including the unique genes [131, 132], namely, ATP citrate lyase (*aclAB* in 1 MAG), *kor* (10 genomes), and fumarate reductase (*frd* in 2 genomes) (Fig. 5d, Dataset S4 Sheet2). Among these unique genes, *aclAB* is the key regulatory enzyme of this cycle [131, 132] but is present in only one genome, Ocean.21468.10 (Dataset S4 Sheet2), which indicates that the rTCA cycles may not be ubiquitous in *Ca.* Cosmopoliota. Based on the above analysis on metabolic potentials, *Ca.* Cosmopoliota may use a wide variety of compounds as electron donors for carbon fixation, such as hydrogen, sulfide, or thiosulfate [132, 133]. Overall, we propose that species in *Ca.* Cosmopoliota are possibly facultative mixotrophs, which maybe one of the explanations for their worldwide distribution.

## Conclusion

In current study, the microbial and metabolic profiles of prokaryotic and fungal communities were investigated using the combination of Illumina and PacBio sequencing. The result demonstrated that the supplement of PacBio long reads for metagenomic analysis significantly improved the contiguity of assemblies, as well as the number and novelty of yielded MAGs. It was revealed that the relative abundance of bacteria was the highest, followed by that of archaea and fungi. Further metabolic reconstruction for recovered MAGs suggested that prokaryotes in mangrove sediment played key roles in nitrogen and sulfur cycling, with versatile capabilities for degrading organic carbons, fermentation, autotrophy, and carbon fixation. Mangrove fungi therein harbor broad metabolic potentials in degradation of various carbohydrate and peptide substrates and potentially participate in organic carbon, nitrogen, and sulfur cycling. Notably, a new bacterial phylum *Candidatus* Cosmopoliota was proposed based on phylogenetic and phylogenomic analyses. Available genomes showed that its members are likely to adopt a versatile lifestyle and utilize various types of organic substrates. In addition, the phylum is capable of anaerobic fermentation and carbon fixation via the WL pathway and the rTCA cycle. Based on the screening of 16S rRNA gene sequences in public databases, the phylum is the most frequently detected in marine and lake sediments, suggesting a possible preference for oxygen-limited environments. Overall, the study highlights the great application potential of third-generation sequencing in metagenomic analysis, provides an overview of microbial community structure, and suggests distinct ecological roles played by diverse microbial groups in mangrove sediments.

### Supplementary Information

**Additional file 1: Supplementary materials.** Results of community structure and metabolic profiles of microbial community in mangrove sediment. And the taxonomic description of new candidate phylum *Candidatus *Cosmopoliota. **Fig. S1.** Statistics of MAGs derived from Illumina assembly, PacBio assembly, and Hybrid assembly. (a) The number and percentage of MAGs under different quality strategies. (b) The Venn diagram shows the distribution of refined MAGs from each assembly. **Fig. S2.** Comparison of draft MAGs derived from Illumina assembly, PacBio assembly, and Hybrid assembly. (a) Comparison of the longest contig and contig number in each draft MAGs derived from three assemblies. (b) Comparison of N50 and contig number in each draft MAGs derived from three assemblies. **Fig. S3.** Average amino acid identity (AAI) comparison of draft MAGs for 12 refined high-quality MAGs. Each of these 12 refined high-quality MAGs have three high-quality (CheckM-completeness ≥ 90%, CheckM-contamination < 5%) and high fastANI similarity (> 99%) draft MAGs derived from three assemblies (Illumina assembly, PacBio assembly, and Hybrid assembly), respectively. **Fig. S4.** Collinearity analyses of draft MAGs for 12 refined high-quality MAGs. Each of these 12 refined high-quality MAGs have three high-quality (CheckM-completeness ≥ 90%, CheckM-contamination < 5%) and high fastANI similarity (> 99%) draft MAGs derived from three assemblies (Illumina assembly, PacBio assembly, and Hybrid assembly), respectively. (a) Genome collinearity of three draft MAGs of each refined MAGs; “Ill” in x-axis represents Illumina, “Hyb” represents Hybrid, and “Pac” represents PacBio; The parts of forward collinearity are displayed in blue color, these of reverse collinearity is in green, and the unmatched regions are in red color. (b) Gene collinearity of three draft MAGs of each refined MAGs; The three long strips consisted of couples of short strips in each subgraph represent three draft MAGs derived from Illumina assembly, Hybrid assembly, and PacBio assembly, respectively; each short strip in long strip represents one contig of the draft MAG; The gray bands connecting two draft MAGs represent the collinearity relationship (> 20 genes) between the genes in two draft MAGs. **Fig. S5.** Genomic collinearity of draft MAGs for 12 refined high-quality MAGs. Each of these 12 refined high-quality MAGs have three high-quality (CheckM-completeness ≥ 90%, CheckM-contamination < 5%) and high fastANI similarity (> 99%) draft MAGs derived from each of three assemblies (Illumina assembly, PacBio assembly, and Hybrid assembly), respectively. The green lines in the graphic are the separation of adjacent contig, which are corresponding to the up x-axis and right y-axis, respectively. While, the bottom x-axis and left y-axis represent the genome location. **Fig. S6.** Gene collinearity of draft MAGs for 12 refined high-quality MAGs. Each of these 12 refined high-quality MAGs have three high-quality (CheckM-completeness ≥ 90%, CheckM-contamination < 5%) and high fastANI similarity (> 99%) draft MAGs derived from each of three assemblies (Illumina assembly, PacBio assembly, and Hybrid assembly), respectively. The green lines in the graphic are the separation of adjacent contig, which are corresponding to the up x-axis and right y-axis, respectively. While, the bottom x-axis and left y-axis represent the genome location. **Fig. S7.** Relative abundance of microbial community and specific genes in different sediment depth. (a) Composition of Prokaryotes, Eukaryotes, Archaea, Bacteria, and Fungi based on 16S rRNA and ITS genes against SILVA database and UNITE database for all eukaryotes, respectively. (b) Composition of Hydrogenases, [NiFe]-Hydrogenases, [FeFe]-Hydrogenases, Carbohydrate-Active Enzymes (CAZymes), and dissimilatory sulfite reductase (*dsr*) genes. **Fig. S8.** Read count of microbial community and specific genes in different sediment depth. Read counts are standardized to CPM (count (read) per million reads). (a) Read count of Prokaryotes, Eukaryotes, Archaea, Bacteria, and Fungi based on 16S rRNA and ITS genes against SILVA database and UNITE database for all eukaryotes, respectively. (b) Read count of Hydrogenases, [NiFe]-Hydrogenases, [FeFe]-Hydrogenases, Carbohydrate-Active Enzymes (CAZymes), and dissimilatory sulfite reductase (*dsr*) genes. **Fig. S9.** Read count of metagenomic reads for specific genes in different sediment depth. Read counts are standardized to CPM (count (read) per million reads). Abbreviation: NiFe/FeFe, [NiFe]-/[FeFe]-hydrogenases; *atpA*, ATP synthase; *coxA*, cytochrome c oxidase; *cyoA*, cytochrome o ubiquinol oxidase; *ccoN*, cytochrome c oxidase; *HCO*, haem-copper oxidase genes (*coxA*, *cyoA* and *ccoN*); *cydA*, cytochrome bd oxidase; *acsB*, acetyl-CoA synthase; *mcrA*, methyl-CoM reductase; *dsr*, dissimilatory sulfite reductase; *sor*, sulfur oxygenase/reductase; *narG*, dissimilatory nitrate reductase; *napA*, periplasmic nitrate reductase; *nir*, dissimilatory nitrite reductase; *nrf*, ammonifying nitrite reductase; *nif*, nitrogenase; *nifH*, nitrogenase iron protein; *rbcL*, ribulose 1,5-bisphosphate carboxylase; *pfor*, pyruvate-ferredoxin oxidoreductase. **Fig. S10.** Glycoside hydrolases (GH) identified by CAZy searches of the MAGs. GH families that contain enzymes that are not specifically involved in degradation were specifically identified by Pfam or EC numbers in the annotations, based on Wrighton et al. 2014. **Fig. S11.** Pathway completeness of all refined MAGs calculated by KEGGDecoder. The pathway completeness is defined as the percentage of core genes of specific pathways identified in each MAG. Complete lists of metabolic genes or pathways can be found in Dataset S2 Sheet7. Detailed gene lists for each pathway indicated can be found at: https://github.com/bjtully/BioData/blob/master/KEGGDecoder/KOALA_definitions.txt. The up row of heatmap shows the MAG completeness, and the bottom raw represent the phylogenetic information of each MAG at phylum level (Archaea in purple and Bacteria in blue). **Fig. S12.** The phylogenetic tree of *Candidatus *Cosmopoliota and the adjacent phyla based on 16S rRNA genes. The genomes of *Ca. *Cosmopoliota are labeled in orange color and bold font. **Fig. S13.** The average amino acid identity (AAI) values between each genome in *Candidatus *Cosmopoliota and the adjacent phyla. The genomes of *Ca. *Cosmopoliota are labeled in orange color and bold font. **Fig. S14.** The average nucleotide identity (ANI) values between the 16S rRNA genes of *Ca. *Cosmopoliota and the adjacent phyla. The genomes of *Ca. Cosmopoliota* are labeled in orange color and bold font.

**Additional file 2: Dataset S1.** Sheet1: Sample information; Sheet2: Stats of raw data; Sheet3: Microbial community estimated based on 16S rRNA (for Prokaryotes) and ITS (for Eukaryotes) by graftM search on SILVA database and blastn search on UNITE database, respectively; Sheet4: Specific genes of prokaryotic community.

**Additional file 3: Dataset S2.** Sheet1: Assembly details of different sequencing methods; Sheet2: Stats of MAGs derived from different sequencing methods and refined MAGs; Sheet3: Draft MAGs used to compare the effects of different assemble methods; Sheet4: Classification, quality, details, coverage depth, and gene number of refined MAGs; Sheet5: Pathway completeness of all refined MAGs calculated by KEGGDecoder; Sheet6: Gene numbers of each pathway annotated by METABOLIC software; Sheet7: Glycoside hydrolases (GH) identified by CAZy searches of the MAGs. GH families that contain enzymes that are not specifically involved in degradation were specifically identified by Pfam or EC numbers in the annotations; Sheet8: Peptidase genes identified against MEROPS database by METABOLIC software; Sheet9: Anaerobic hydrocarbon degradation genes annotated by METABOLIC software.

**Additional file 4: Dataset S3.** Sheet1: The metabolic characteristics of the major fungal groups annotated against KEGG; Sheet2: Detail information of the carbohydrate-active enzyme family (CAZymes) detected in major fungal groups; Abbreviations: GH, glycosidases or glycosyl hydrolases; PL, polysaccharide lyases; CE, carbohydrate esterases; GT, glycosyltransferases; AA, auxiliary activities; CBM, carbohydrate binding modules; Sheet3: Detail information of the peptidase family genes detected in major fungal groups; Abbreviations: Aspartic (A), Cysteine (C), Metallo (M), Mixed (P), Serine (S) and Threonine (T).

**Additional file 5: Dataset S4.** Sheet1: Detail information for MAGs belong to *Candidatus *Cosmopoliota; Sheet2: The metabolic characteristics of *Ca. *Cosmopoliota, annotated against KEGG database; Sheet3: Detail information of CAZymes encoded in *Ca. *Cosmopoliota; Abbreviations: GH, glycosidases or glycosyl hydrolases; PL, polysaccharide lyases; CE, carbohydrate esterases; GT, glycosyltransferases; AA, auxiliary activities; CBM, carbohydrate binding modules; Sheet4: Detail information of peptidase encoded in *Ca. *Cosmopoliota; Abbreviations: Aspartic (A), Cysteine (C), Metallo (M), Mixed (P), Serine (S) and Threonine (T); Sheet5: Gene functions of each MAG annotated by metabolic software; Sheet6: Information of sequences belonging to *Ca. *Cosmopoliota (identity > 83%) retrieved from GenBank; Sheet7: SRA samples with sequences belonging to *Ca. *Cosmopoliota (identity > 83%) retrieved from Microbe Atlas Project by MAPseq.

## Data Availability

Raw reads of the three mangrove sediment metagenomes have been deposited in NODE (The National Omics Data Encyclopedia, https://www.biosino.org/node/) under the project ID OEP004054 (for Illumina metagenomes) and OEP004055 (for PacBio metagenomes). The MAGs have been deposited in eLMSG (an eLibrary of Microbial Systematics and Genomics, https://www.biosino.org/elmsg/index) under the accession number LMSG_G000013458.1-LMSG_G000014019.1 (accession link in related document “private_link_for_reviewer.xlsx”).
