# Supplementary material for: Long-read assembled metagenomic approaches improve our understanding on metabolic potentials of microbial community in mangrove sediments
Source: Microbiome. 2023 Aug 23;11:188. doi: 10.1186/s40168-023-01630-x (PMC10464287; doi:10.1186/s40168-023-01630-x)
Supplement: Supplementary file 2 — Additional file 1: Supplementary materials. Results of community structure and metabolic profiles of microbial community in mangrove sediment. And the taxonomic description of new candidate phylum Candidatus Cosmopoliota. Fig. S1. Statistics of MAGs derived from Illumina assembly, PacBio assembly, and Hybrid assembly. (a) The number and percentage of MAGs under different quality strategies. (b) The Venn diagram shows the distribution of refined MAGs from each assembly. Fig. S2. Comparison of draft MAGs derived from Illumina assembly, PacBio assembly, and Hybrid assembly. (a) Comparison of the longest contig and contig number in each draft MAGs derived from three assemblies. (b) Comparison of N50 and contig number in each draft MAGs derived from three assemblies. Fig. S3. Average amino acid identity (AAI) comparison of draft MAGs for 12 refined high-quality MAGs. Each of these 12 refined high-quality MAGs have three high-quality (CheckM-completeness ≥ 90%, CheckM-contamination < 5%) and high fastANI similarity (> 99%) draft MAGs derived from three assemblies (Illumina assembly, PacBio assembly, and Hybrid assembly), respectively. Fig. S4. Collinearity analyses of draft MAGs for 12 refined high-quality MAGs. Each of these 12 refined high-quality MAGs have three high-quality (CheckM-completeness ≥ 90%, CheckM-contamination < 5%) and high fastANI similarity (> 99%) draft MAGs derived from three assemblies (Illumina assembly, PacBio assembly, and Hybrid assembly), respectively. (a) Genome collinearity of three draft MAGs of each refined MAGs; “Ill” in x-axis represents Illumina, “Hyb” represents Hybrid, and “Pac” represents PacBio; The parts of forward collinearity are displayed in blue color, these of reverse collinearity is in green, and the unmatched regions are in red color. (b) Gene collinearity of three draft MAGs of each refined MAGs; The three long strips consisted of couples of short strips in each subgraph represent three draft MAGs derived from Illumina assemb [file 40168_2023_1630_MOESM1_ESM.docx]

**Supplementary Information**

**Long-Read Assembled Metagenomic Approaches Improve Our Understanding on Metabolic Potentials of Microbial Community in Mangrove Sediments**

Zhi-Feng Zhang^a,b, c^, Li-Rui Liu^a,b^, Yue-Ping Pan^a,b^, Jie Pan^a,b^, Meng Li^a,b,^*

a. Archaeal Biology Center, Institute for Advanced Study, Shenzhen University, Shenzhen, China

b. Shenzhen Key Laboratory of Marine Microbiome Engineering, Institute for Advanced Study, Shenzhen University, Shenzhen, China

c. Southern Marine Science and Engineering Guangdong Laboratory (Guangzhou), Guangzhou, 511458, China (Current address)

*Correspondence: limeng848@szu.edu.cn

**List of Supplementary Information**

**1. Supplementary Materials:** Results of community structure and metabolic profiles of microbial community in mangrove sediment. And the taxonomic description of new candidate phylum *Candidatus* Cosmopoliota.

**2. Supplementary Figures:**

**Fig. S1** Statistics of MAGs derived from Illumina assembly, PacBio assembly, and Hybrid assembly. (a) The number and percentage of MAGs under different quality strategies. (b) The Venn diagram shows the distribution of refined MAGs from each assembly.

**Fig. S2** Comparison of draft MAGs derived from Illumina assembly, PacBio assembly, and Hybrid assembly. (a) Comparison of the longest contig and contig number in each draft MAGs derived from three assemblies. (b) Comparison of N50 and contig number in each draft MAGs derived from three assemblies.

**Fig. S3** Average amino acid identity (AAI) comparison of draft MAGs for 12 refined high-quality MAGs. Each of these 12 refined high-quality MAGs have three high-quality (CheckM-completeness ≥ 90%, CheckM-contamination < 5%) and high fastANI similarity (> 99%) draft MAGs derived from three assemblies (Illumina assembly, PacBio assembly, and Hybrid assembly), respectively.

**Fig. S4** Collinearity analyses of draft MAGs for 12 refined high-quality MAGs. Each of these 12 refined high-quality MAGs have three high-quality (CheckM-completeness ≥ 90%, CheckM-contamination < 5%) and high fastANI similarity (> 99%) draft MAGs derived from three assemblies (Illumina assembly, PacBio assembly, and Hybrid assembly), respectively. (a) Genome collinearity of three draft MAGs of each refined MAGs; “Ill” in x-axis represents Illumina, “Hyb” represents Hybrid, and “Pac” represents PacBio; The parts of forward collinearity are displayed in blue color, these of reverse collinearity is in green, and the unmatched regions are in red color. (b) Gene collinearity of three draft MAGs of each refined MAGs; The three long strips consisted of couples of short strips in each subgraph represent three draft MAGs derived from Illumina assembly, Hybrid assembly, and PacBio assembly, respectively; each short strip in long strip represents one contig of the draft MAG; The gray bands connecting two draft MAGs represent the collinearity relationship (> 20 genes) between the genes in two draft MAGs.

**Fig. S5** Genomic collinearity of draft MAGs for 12 refined high-quality MAGs. Each of these 12 refined high-quality MAGs have three high-quality (CheckM-completeness ≥ 90%, CheckM-contamination < 5%) and high fastANI similarity (> 99%) draft MAGs derived from each of three assemblies (Illumina assembly, PacBio assembly, and Hybrid assembly), respectively. The green lines in the graphic are the separation of adjacent contig, which are corresponding to the up x-axis and right y-axis, respectively. While, the bottom x-axis and left y-axis represent the genome location.

**Fig. S6** Gene collinearity of draft MAGs for 12 refined high-quality MAGs. Each of these 12 refined high-quality MAGs have three high-quality (CheckM-completeness ≥ 90%, CheckM-contamination < 5%) and high fastANI similarity (> 99%) draft MAGs derived from each of three assemblies (Illumina assembly, PacBio assembly, and Hybrid assembly), respectively. The green lines in the graphic are the separation of adjacent contig, which are corresponding to the up x-axis and right y-axis, respectively. While, the bottom x-axis and left y-axis represent the genome location.

**Fig. S7** Relative abundance of microbial community and specific genes in different sediment depth. (a) Composition of Prokaryotes, Eukaryotes, Archaea, Bacteria, and Fungi based on 16S rRNA and ITS genes against SILVA database and UNITE database for all eukaryotes, respectively. (b) Composition of Hydrogenases, [NiFe]-Hydrogenases, [FeFe]-Hydrogenases, Carbohydrate-Active Enzymes (CAZymes), and dissimilatory sulfite reductase (*dsr*) genes.

**Fig. S8** Read count of microbial community and specific genes in different sediment depth. Read counts are standardized to CPM (count (read) per million reads). (a) Read count of Prokaryotes, Eukaryotes, Archaea, Bacteria, and Fungi based on 16S rRNA and ITS genes against SILVA database and UNITE database for all eukaryotes, respectively. (b) Read count of Hydrogenases, [NiFe]-Hydrogenases, [FeFe]-Hydrogenases, Carbohydrate-Active Enzymes (CAZymes), and dissimilatory sulfite reductase (*dsr*) genes.

**Fig. S9** Read count of metagenomic reads for specific genes in different sediment depth. Read counts are standardized to CPM (count (read) per million reads). Abbreviation: NiFe/FeFe, [NiFe]-/[FeFe]-hydrogenases; *atpA*, ATP synthase; *coxA*, cytochrome c oxidase; *cyoA*, cytochrome o ubiquinol oxidase; *ccoN*, cytochrome c oxidase; *HCO*, haem-copper oxidase genes (*coxA*, *cyoA* and *ccoN*); *cydA*, cytochrome bd oxidase; *acsB*, acetyl-CoA synthase; *mcrA*, methyl-CoM reductase; *dsr*, dissimilatory sulfite reductase; *sor*, sulfur oxygenase/reductase; *narG*, dissimilatory nitrate reductase; *napA*, periplasmic nitrate reductase; *nir*, dissimilatory nitrite reductase; *nrf*, ammonifying nitrite reductase; *nif*, nitrogenase; *nifH*, nitrogenase iron protein; *rbcL*, ribulose 1,5-bisphosphate carboxylase; *pfor*, pyruvate-ferredoxin oxidoreductase.

**Fig. S10** Glycoside hydrolases (GH) identified by CAZy searches of the MAGs. GH families that contain enzymes that are not specifically involved in degradation were specifically identified by Pfam or EC numbers in the annotations, based on Wrighton et al. 2014.

**Fig. S11** Pathway completeness of all refined MAGs calculated by KEGGDecoder. The pathway completeness is defined as the percentage of core genes of specific pathways identified in each MAG. Complete lists of metabolic genes or pathways can be found in Dataset S2 Sheet7. Detailed gene lists for each pathway indicated can be found at: https://github.com/bjtully/BioData/blob/master/KEGGDecoder/KOALA_definitions.txt. The up row of heatmap shows the MAG completeness, and the bottom raw represent the phylogenetic information of each MAG at phylum level (Archaea in purple and Bacteria in blue).

**Fig. S12** The phylogenetic tree of *Candidatus* Cosmopoliota and the adjacent phyla based on 16S rRNA genes. The genomes of *Ca.* Cosmopoliota are labeled in orange color and bold font.

**Fig. S13** The average amino acid identity (AAI) values between each genome in *Candidatus* Cosmopoliota and the adjacent phyla. The genomes of *Ca.* Cosmopoliota are labeled in orange color and bold font.

**Fig. S14** The average nucleotide identity (ANI) values between the 16S rRNA genes of *Ca.* Cosmopoliota and the adjacent phyla. The genomes of *Ca. Cosmopoliota* are labeled in orange color and bold font.

**1. Supplementary Materials**

**Results and discussion**

**1.1 Depth-stratified prokaryotic and eukaryotic communities**

Prokaryotic and eukaryotic profiles were determined based on 16S rRNA (for prokaryotes) and internal transcribed spacers (ITS, for Eukaryotes) by graftM^1^ search on SILVA database^2^ and BLASTn^3^ search on UNITE database^4^, respectively (Fig. S7a, Fig. S8a and Dataset S1 sheet3). Overall, the microbial composition was consistent with previous investigation^5^, that prokaryotic abundance was much higher than eukaryotic abundance, both in surface and subsurface sediment. To be exact, the CPM (count per million reads) of prokaryotes was 8.5 to 11.0 times higher than that of eukaryotes from 0–10 cm depth sediment to 20–30 cm depth sediment. Most of predicted eukaryotes were unknown, and fungi was the most abundant eukaryotic kingdom, with motazoa, stramenopila, alveolata, viridiplantae, protista, and rhizaria following. The CPM of eukaryotes, fungi, and motazoa was higher in surface sediment than subsurface (Fig. 3a, S7a, S8a, and Dataset S1 Sheet3). For Prokaryotes, the CPM was also slightly decreasing from surface to subsurface sediment. However, the distribution of archaea and bacteria were totally different. The relative abundance of archaea was greatly increasing along sediment depth, which was about three times higher in 20-30 cm depth sediment than that in surface sediment. In contrary, the abundance of bacteria was decreasing along sediment depth (Fig. S7a, Fig. S8a, and Dataset S1 Sheet3). Although the CPM of eukaryotes and prokaryotes were changed along sediment depth, the Shannon indices were nearly constant (3.91±0.15 in 0–10 cm depth, and 3.86±0.11 in 10–30 cm depth).

For all microorganisms, fungi had the lowest abundance and percentage (CPM: 11.3–16.6, percentage: 3.2%–4.4%), and bacteria were the most abundant (CPM: 256.4–333.4, percentage: 73.2%–87.5%). In archaea, *Euryarchaeota*, *Woesearchaeota*, *Bathyarchaeota*, Asgard archaea were predominant. Consistent with the investigation of Zhang et al.^6^, *Euryarchaeota*, *Bathyarchaeota*, and Asgard archaea were much more abundant in subsurface than surface sediment, while, *Woesearchaeota* was more abundant in surface sediment. *Proteobacteria*, followed by *Chloroflexi*, *Desulfobacterota*, *Bacteroidota*, and *Planctomycetota* were the most abundant bacterial phyla. Among the top ten predominant bacterial phyla, only two phyla, *Chloroflexi* and *Planctomycetota*, were more abundant in subsurface sediment than in surface sediment. Similarly, the relative abundances of archaeal phyla *Euryarchaeota*, *Bathyarchaeota*, and Asgard archaea, and bacterial phyla *Chloroflexi* and *Planctomycetota* were possibly positively correlated with pH, nitrate and total sulfur (TS), while that of *Woesearchaeota* and most bacterial phyla might be positively correlated with total organic carbon (TOC), total carbon (TC), and total nitrogen (TN). For fungi, *Ascomycota* and *Basidiomycota* were the most abundant, and both of their abundance were decreasing along sediment. Meanwhile, environmental variable analyses showed that the abundance of fungi, *Ascomycota*, and *Basidiomycota* might be positively correlated with TOC, TC, and TN, but negatively correlated with pH, nitrate and TS (Fig. 3a, Fig. S7a, Fig. S8a, and Dataset S1 sheet3). The observations provided supporting for previous composition studies relied on specific marker genes^6-8^. In summary, bacteria are the predominant microorganism, and both bacteria and fungi are more abundant in surface sediment, while *Archaea* were more abundant in subsurface sediment, and environmental variables such as TC, TOC, TN, nitrate, and TS were possible influencing factors on microbial abundance.

**1.2 *Depth-stratified metabolic function of prokaryotic community***

The overall metabolic potential of prokaryotes in sediment was predicated by aligning filtered Illumina reads to TIGRFAMs^9^ and Pfam^10^ databases using DIAMOND BLASTx^11^ (Fig. S9, and Dataset S1 Sheet4). *pfor* (pyruvate-ferredoxin oxidoreductase), *NiFe* ([NiFe]-hydrogenases), *atpA* (ATP synthase), *coxA* (Cytochrome c oxidase), and *dsr* (dissimilatory sulfite reductase) were the most abundant genes in sediment, and all of them were more abundant in surface than in subsurface sediment, and were possibly positively correlated with TOC, TC, and TN. Consistent with the overall decreasing distribution of microbes, most of the representative genes were decreasing in subsurface sediment comparing with surface sediment, except for *mcrA* (methyl-CoM reductase), *sor* (sulfur oxygenase/reductase), and *nifH* (nitrogenase), which might be negatively correlated with TOC, TC, and TN. *mcrA* and *sor*, core genes for methanogenesis and sulfur metabolism respectively, were more abundant in 10-30cm sediment, indicating that methanogenesis and sulfur metabolism might be more activate in subsurface sediment (Fig. S9 and Dataset S1 sheet4). It may could be explained by the increasing abundant of the main methanogenesis microbes in subsurface sediment (Dataset S1 sheet3), such as *Euryarchaeota* including *Methanobacteriales*, *Methanomassiliicoccales*, *Methanomicrobiales*, *Methanofastidiosales*^12^. Interestingly, the abundance of *nifH*, the key enzyme in nitrogen fixation, were consistent in 0–10cm and 20–30cm depth sediment.

To further investigate the composition and distribution of several specific genes, such as hydrogenases, Carbohydrate-Active Enzymes (CAZymes), and *dsr*, raw metagenomic sequences were searched against customed databases separately^13-15^. In total, 990,729 hydrogenase sequences were identified from the metagenomic short reads, with the abundance slightly decreasing from surface sediment to subsurface sediment, in which, the concentrations of TOC, TC, and TN were consistently decreasing (Fig. S8b, Dataset S1 Sheet1 and Sheet4). The percentages of *NiFe* and *FeFe* were almost the same, ca. 50% respectively, and few was [*Fe*]-hydrogenases (less than 0.01%) (Fig. S7b and Dataset S1 Sheet4). The abundance of *NiFe* was decreasing along sediment depth, while that of *NiFe* was inverse. Although the abundance was decreasing overall, the subgroups of *NiFe* were different. For example, *NiFe* group 3c and 1a were more abundant in subsurface than in surface sediment. These two hydrogenase groups are capable to bifurcate electrons from H_2_ to heterodisulfide and Fd_ox_ in methanogens, and participate in sulfate, metal, and organohalide respiration respectively^13^, indicating that these metabolic pathways might be more abundant in subsurface sediment. In contrast, the abundance of *NiFe* group 3d, 1b, 1d, and 1e were higher in surface than subsurface sediment. Meanwhile, the abundant of *FeFe* group A3, which reversibly bifurcates electrons from H_2_ to NAD and Fd_ox_ in anaerobic bacteria^13^, was increased with sediment depth decreasing. It indicated that anaerobic hydrogen-consuming bacteria might be more abundant in deep mangrove sediment (Fig. S7b, Fig. S8b, and Dataset S1 Sheet4).

We classified more than 13.6 million short sequences for CAZymes, with CPM as high as 10,670 to 8,820 from surface sediment to subsurface sediment, suggesting a possibly high capability of breakdown, biosynthesis or modification of carbohydrates and glycoconjugates in mangrove sediment^14^ (Fig. S8b, and Dataset S1 Sheet4). Genes of GH (Glycoside Hydrolases) module were most abundant, both in surface and subsurface sediment. Five of the six modules were decreasing along sediment depth, except for PL (Polysaccharide Lyases), which was more abundant in subsurface sediment in contrast. Based on custom *dsrABD* database, 86,079 short sequences were assigned as *dsr* genes, including *dsrA*, *dsrB* and *dsrD*. All these three genes were decreasing along the sediment depth (Fig. S8b, and Dataset S1 Sheet4).

**1.3** **New candidate phylum *Candidatus* Cosmopoliota**

**Taxonomic description**

**Description of *Candidatus* Cosmopoliota phy. nov.**

*Candidatus* Cosmopoliota (Cosmopoli’ota, N.L fem. n. *Candidatus* *Cosmopolia*, type genus of the order *Candidatus* *Cosmopoliales*, the type order of the class *Candidatus Cosmopolitia*, the type class of the phylum; N.L. neut. n. suff. *-ota*, ending to denote a phylum; N.L. neut. n. *Candidatus Cosmopoliota*, the phylum of the class *Candidatus Cosmopolitia*)

**Description of *Candidatus* *Cosmopolitia* cla. nov.**

*Candidatus Cosmopolitia* (Cosmopoli’tia, N.L fem. n. *Candidatus* *Cosmopolia*, type genus of the order *Candidatus* *Cosmopoliales*, the type order of the class; N.L. neut. n. suff. *-ia*, ending to denote a class; N.L. neut. pl. n. *Candidatus Cosmopolitia*, the class of the order *Candidatus Cosmopoliales*)

**Description of *Candidatus* *Cosmopoliales* ord. nov.**

*Candidatus Cosmopoliales* (Cosmopoli.a’les, N.L fem. n. *Candidatus* *Cosmopolia*, type genus of the order; L. fem. pl. suff. *-ales*, ending to denote an order; N.L. fem. pl. n. *Candidatus Cosmopoliales*, the order of *Candidatus* *Cosmopolia*)

**Description of *Candidatus* *Cosmopoliaceae* fam. nov.**

*Candidatus* *Cosmopoliaceae* (Cosmopoli.a.ce’ae, N.L. fem. n. *Candidatus* *Cosmopolia*, type genus of the family, L. fem. pl. suff. *-aceae*, ending to denote a family; N.L. fem. pl. n. *Candidatus* *Cosmopoliaceae*, the family of *Candidatus* *Cosmopolia*)

**Description of *Candidatus* *Cosmopolia* gen. nov.**

*Candidatus* *Cosmopolia* (Cosmopoli’a, N.L. fem. n. *Candidatus* *Cosmopoli* from Gr. n. cosmopolitan, referring the worldwide distribution of the bacterium)

**Description of *Candidatus* *Cosmopolia* *futianensis* sp. nov.**

*Candidatus* *Cosmopolia* *futianensis* (futian’ensis N.L. masc. adj. pertaining to the Futian Mangrove National Nature Reserve of China, where the type material of this species was obtained). Type material of *Candidatus* *Cosmopolia* *futianensis* is the genome Bin.344 representing. The genome Bin.344 represents a MAG consisting of 4.8 Mbps in 37 contigs with an estimated completeness of 95.5%, an estimated contamination of 1.1%, a GC content of 42.5%, a 16S rRNA gene, a 5S rRNA gene, and 45 tRNAs.

**References**

1. Boyd JA, Woodcroft BJ, Tyson GW. GraftM: a tool for scalable, phylogenetically informed classification of genes within metagenomes. *Nucleic Acids Res* **46**, e59 (2018).

2. Quast C*, et al.* The SILVA ribosomal RNA gene database project: improved data processing and web-based tools. *Nucleic Acids Res* **41**, D590-596 (2013).

3. Camacho C*, et al.* BLAST+: architecture and applications. *BMC Bioinf* **10**, 421 (2009).

4. Nilsson RH*, et al.* The UNITE database for molecular identification of fungi: handling dark taxa and parallel taxonomic classifications. *Nucleic Acids Res* **47**, D259–D264 (2019).

5. Simões MF*, et al.* Soil and Rhizosphere Associated Fungi in Gray Mangroves (Avicennia marina) from the Red Sea--A Metagenomic Approach. *Genom Proteom Bioinf* **13**, 310-320 (2015).

6. Zhang ZF, Pan J, Pan YP, Li M. Biogeography, Assembly Patterns, Driving Factors, and Interactions of Archaeal Community in Mangrove Sediments. *mSystems*, e0138120 (2021).

7. Zhang CJ*, et al.* Prokaryotic Diversity in Mangrove Sediments across Southeastern China Fundamentally Differs from That in Other Biomes. *mSystems* **4**, (2019).

8. Zhang ZF, Pan YP, Liu Y, Li M. High-Level Diversity of Basal Fungal Lineages and the Control of Fungal Community Assembly by Stochastic Processes in Mangrove Sediments. *Appl Environ Microbiol* **87**, e0092821 (2021).

9. Haft DH, Selengut JD, Richter RA, Harkins D, Basu MK, Beck E. TIGRFAMs and Genome Properties in 2013. *Nucleic Acids Res* **41**, D387-395 (2013).

10. Mistry J*, et al.* Pfam: The protein families database in 2021. *Nucleic Acids Res* **49**, D412-D419 (2021).

11. Buchfink B, Xie C, Huson DH. Fast and sensitive protein alignment using DIAMOND. *Nat Methods* **12**, 59-60 (2015).

12. Evans PN*, et al.* An evolving view of methane metabolism in the Archaea. *Nat Rev Microbiol* **17**, 219-232 (2019).

13. Sondergaard D, Pedersen CN, Greening C. HydDB: A web tool for hydrogenase classification and analysis. *Sci Rep* **6**, 34212 (2016).

14. Zhang H*, et al.* dbCAN2: a meta server for automated carbohydrate-active enzyme annotation. *Nucleic Acids Res* **46**, W95-W101 (2018).

15. Anantharaman K*, et al.* Expanded diversity of microbial groups that shape the dissimilatory sulfur cycle. *ISME J* **12**, 1715-1728 (2018).

**2. Supplementary Figures:**


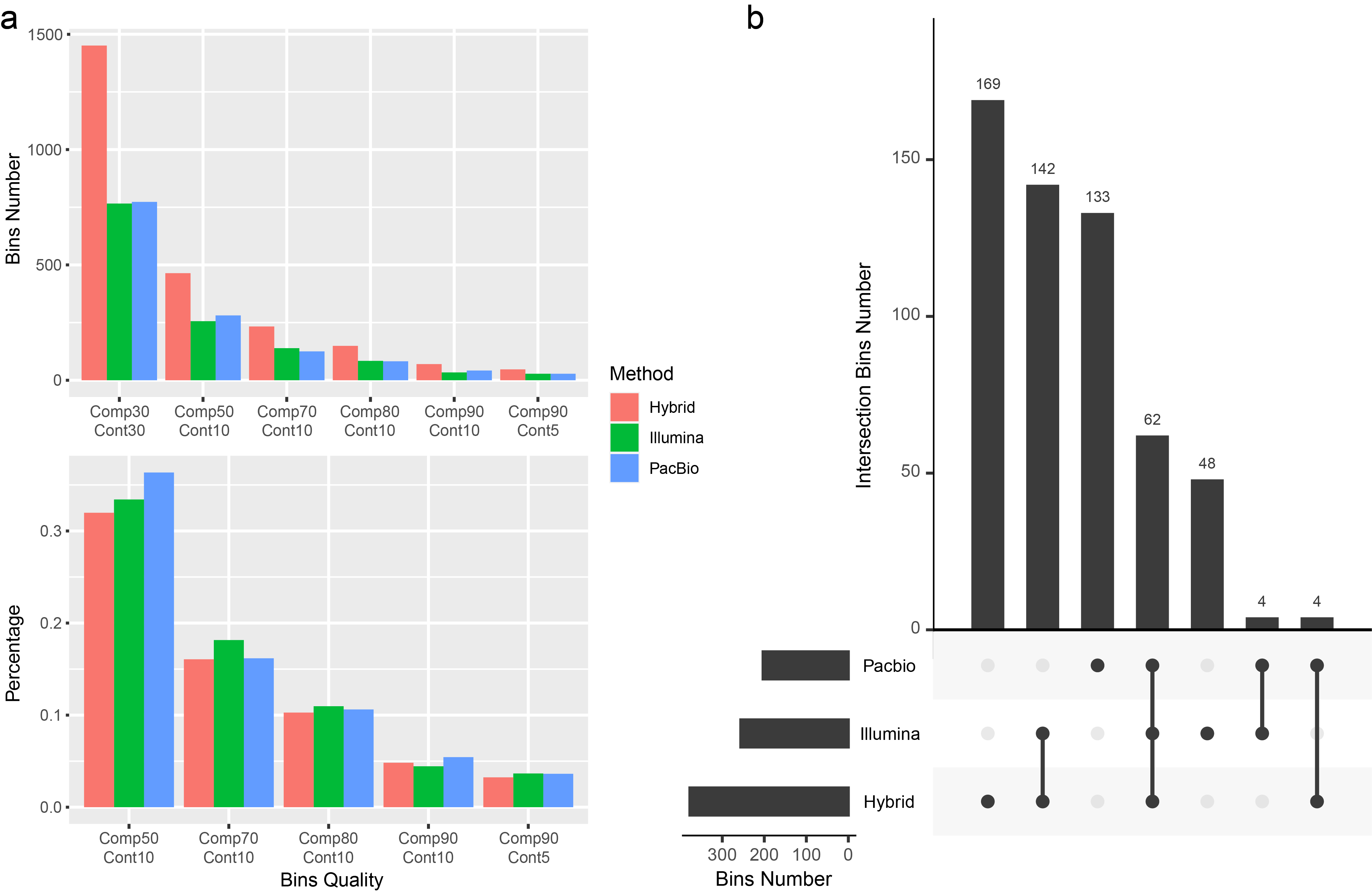


**Fig. S1** Statistics of MAGs derived from Illumina assembly, PacBio assembly, and Hybrid assembly. (a) The number and percentage of MAGs under different quality strategies. (b) The Venn diagram shows the distribution of refined MAGs from each assembly.


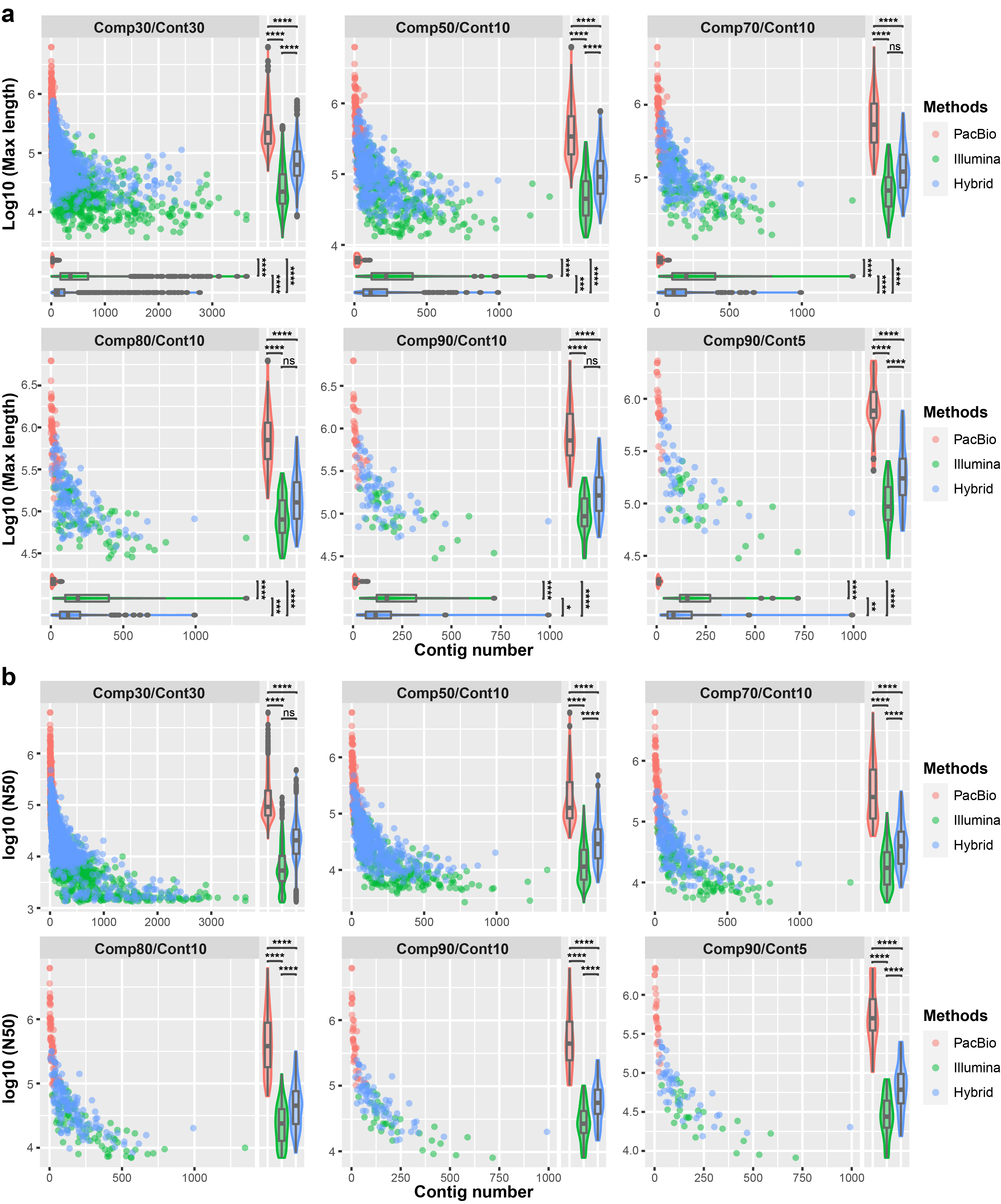


**Fig. S2** Comparison of draft MAGs derived from Illumina assembly, PacBio assembly, and Hybrid assembly. (a) Comparison of the longest contig and contig number in each draft MAGs derived from three assemblies. (b) Comparison of N50 and contig number in each draft MAGs derived from three assemblies.


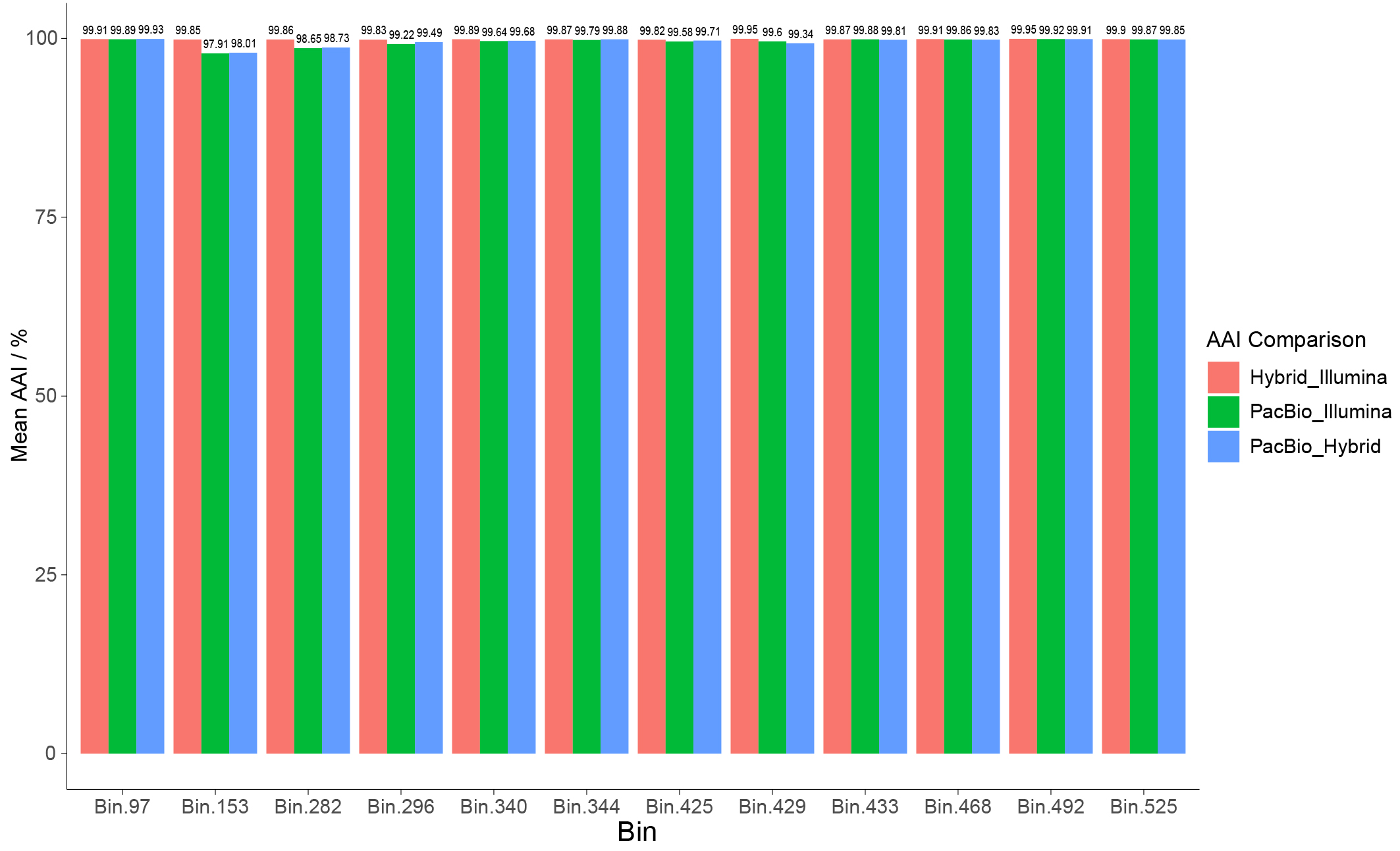
**Fig. S3** Average amino acid identity (AAI) comparison of draft MAGs for 12 refined high-quality MAGs. Each of these 12 refined high-quality MAGs have three high-quality (CheckM-completeness ≥ 90%, CheckM-contamination < 5%) and high fastANI similarity (> 99%) draft MAGs derived from three assemblies (Illumina assembly, PacBio assembly, and Hybrid assembly), respectively.


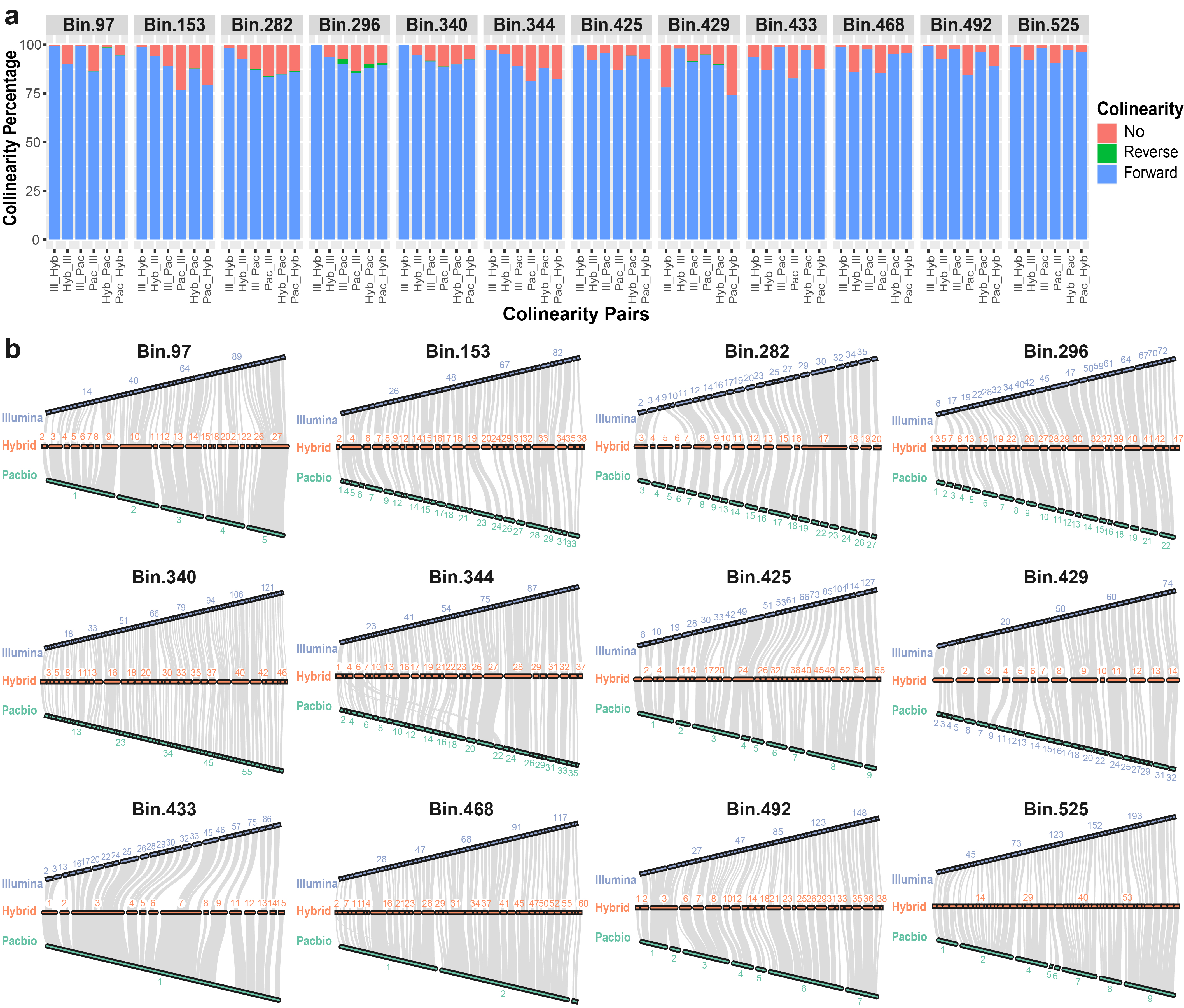
**Fig. S4** Collinearity analyses of draft MAGs for 12 refined high-quality MAGs. Each of these 12 refined high-quality MAGs have three high-quality (CheckM-completeness ≥ 90%, CheckM-contamination < 5%) and high fastANI similarity (> 99%) draft MAGs derived from three assemblies (Illumina assembly, PacBio assembly, and Hybrid assembly), respectively. (a) Genome collinearity of three draft MAGs of each refined MAGs; “Ill” in x-axis represents Illumina, “Hyb” represents Hybrid, and “Pac” represents PacBio; The parts of forward collinearity are displayed in blue color, these of reverse collinearity is in green, and the unmatched regions are in red color. (b) Gene collinearity of three draft MAGs of each refined MAGs; The three long strips consisted of couples of short strips in each subgraph represent three draft MAGs derived from Illumina assembly, Hybrid assembly, and PacBio assembly, respectively; each short strip in long strip represents one contig of the draft MAG; The gray bands connecting two draft MAGs represent the collinearity relationship (> 20 genes) between the genes in two draft MAGs.


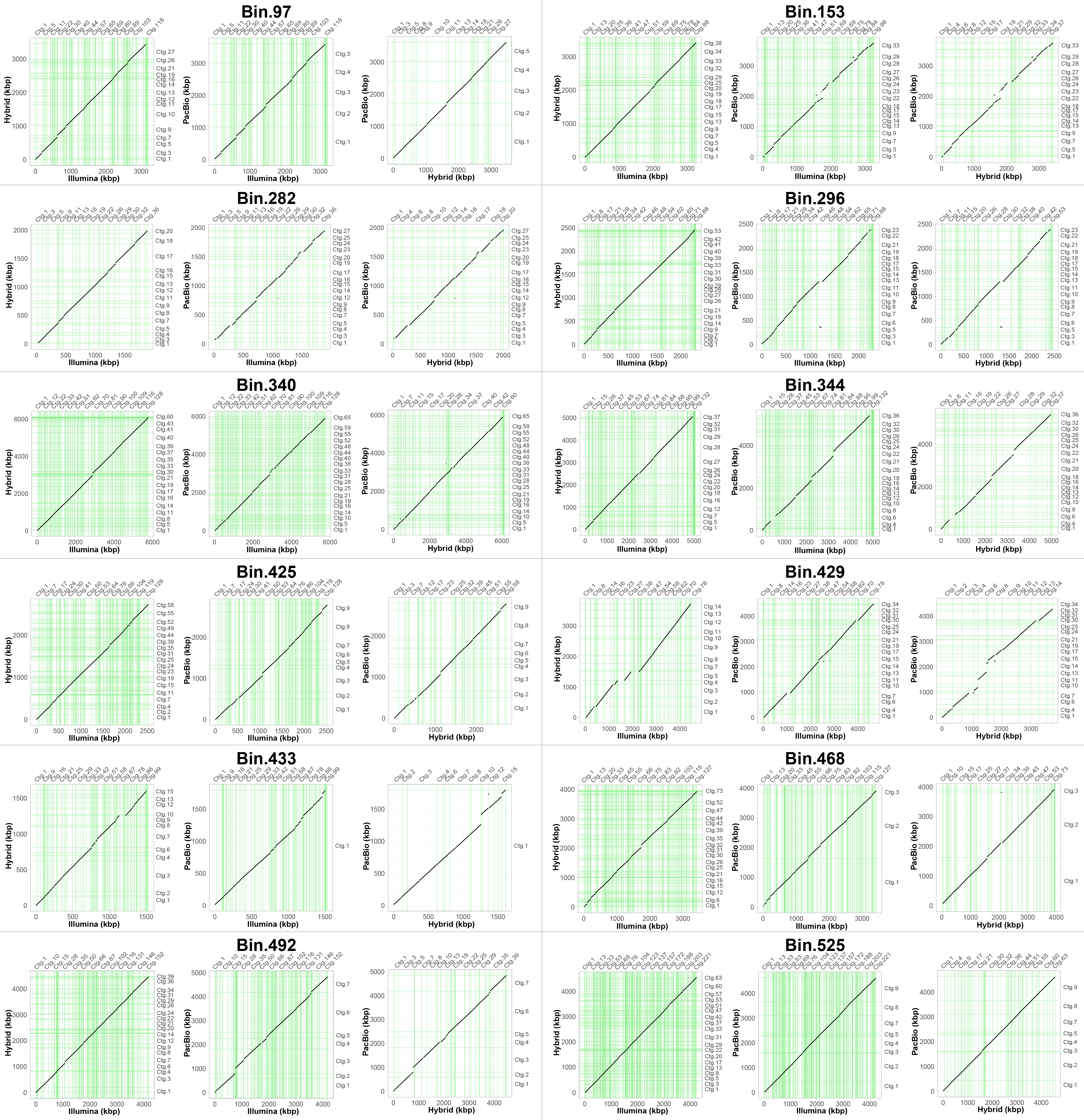
**Fig. S5** Genomic collinearity of draft MAGs for 12 refined high-quality MAGs. Each of these 12 refined high-quality MAGs have three high-quality (CheckM-completeness ≥ 90%, CheckM-contamination < 5%) and high fastANI similarity (> 99%) draft MAGs derived from each of three assemblies (Illumina assembly, PacBio assembly, and Hybrid assembly), respectively. The green lines in the graphic are the separation of adjacent contig, which are corresponding to the up x-axis and right y-axis, respectively. While, the bottom x-axis and left y-axis represent the genome location.


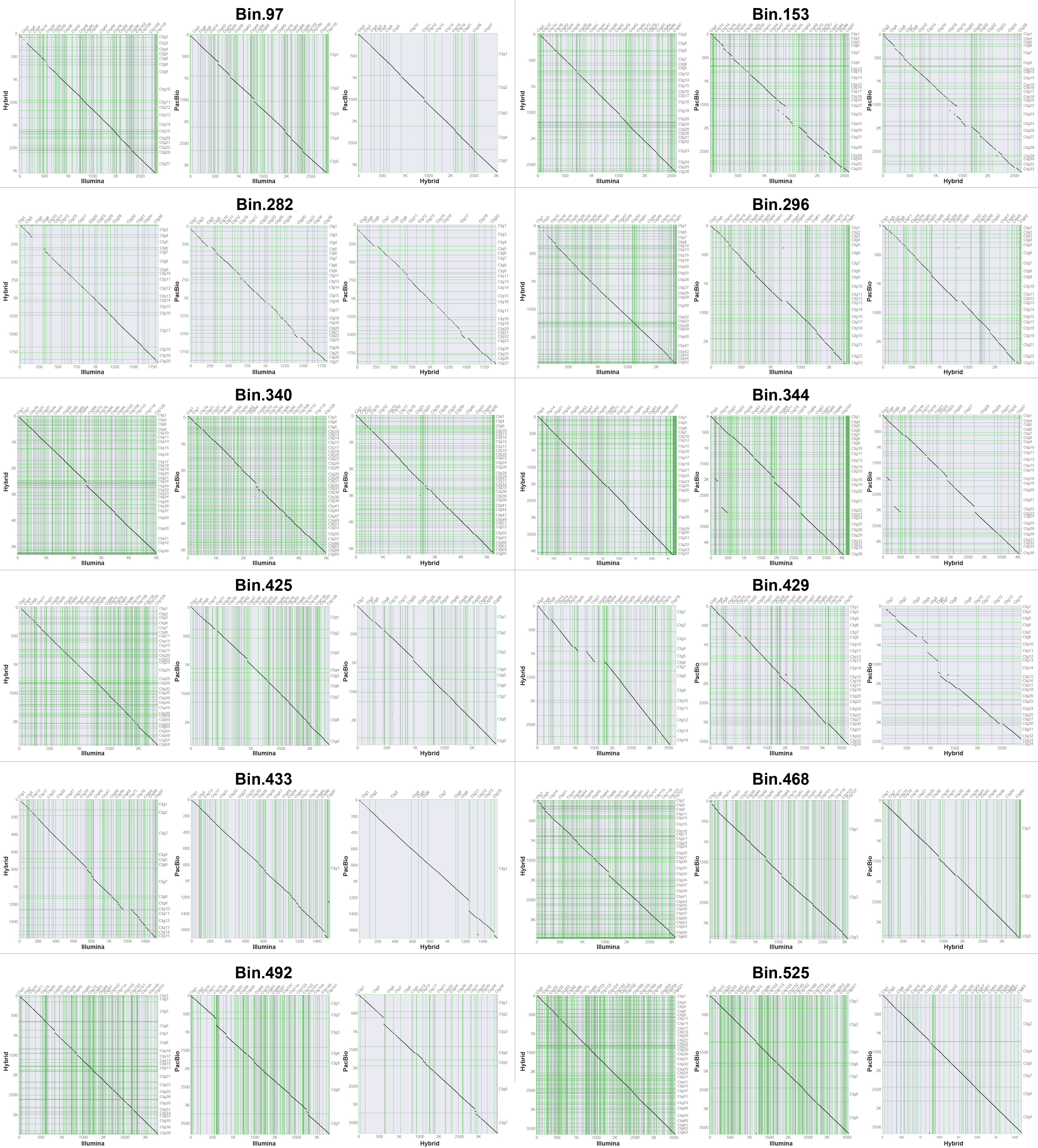
**Fig. S6** Gene collinearity of draft MAGs for 12 refined high-quality MAGs. Each of these 12 refined high-quality MAGs have three high-quality (CheckM-completeness ≥ 90%, CheckM-contamination < 5%) and high fastANI similarity (> 99%) draft MAGs derived from each of three assemblies (Illumina assembly, PacBio assembly, and Hybrid assembly), respectively. The green lines in the graphic are the separation of adjacent contig, which are corresponding to the up x-axis and right y-axis, respectively. While, the bottom x-axis and left y-axis represent the genome location.


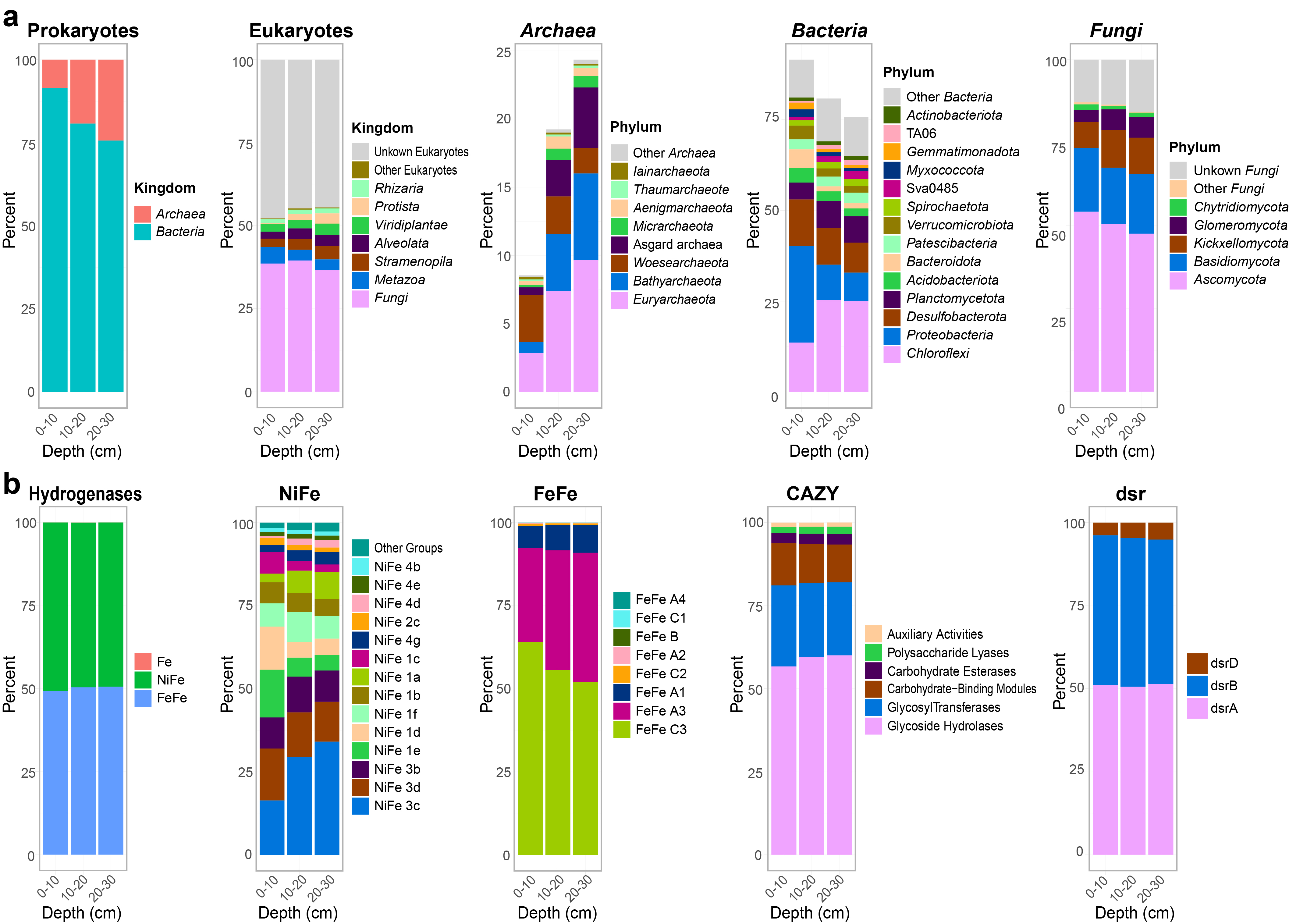
**Fig. S7** Relative abundance of microbial community and specific genes in different sediment depth. (a) Composition of Prokaryotes, Eukaryotes, Archaea, Bacteria, and Fungi based on 16S rRNA and ITS genes against SILVA database and UNITE database for all eukaryotes, respectively. (b) Composition of Hydrogenases, [NiFe]-Hydrogenases, [FeFe]-Hydrogenases, Carbohydrate-Active Enzymes (CAZymes), and dissimilatory sulfite reductase (*dsr*) genes.


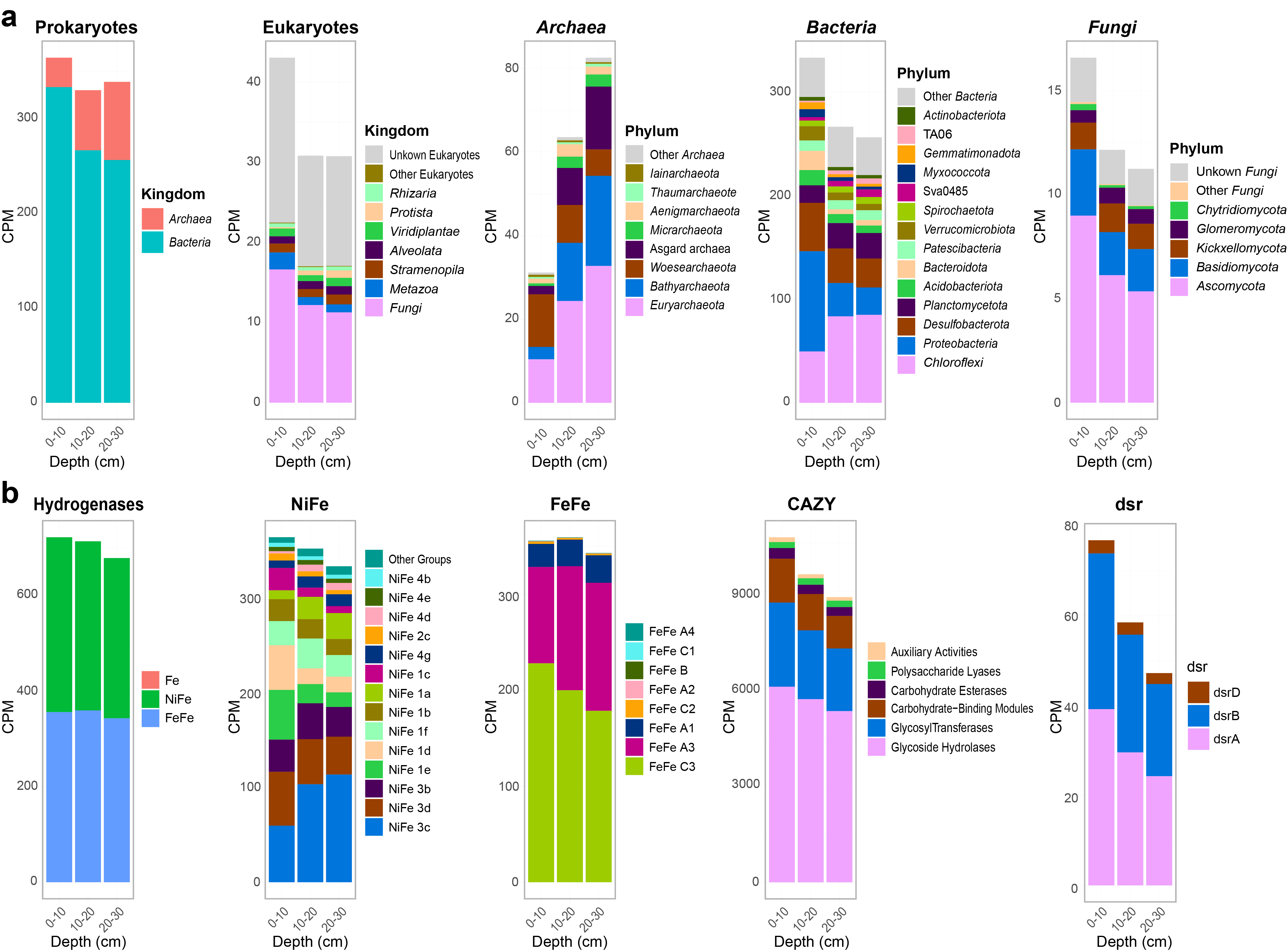
**Fig. S8** Read count of microbial community and specific genes in different sediment depth. Read counts are standardized to CPM (count (read) per million reads). (a) Read count of Prokaryotes, Eukaryotes, Archaea, Bacteria, and Fungi based on 16S rRNA and ITS genes against SILVA database and UNITE database for all eukaryotes, respectively. (b) Read count of Hydrogenases, [NiFe]-Hydrogenases, [FeFe]-Hydrogenases, Carbohydrate-Active Enzymes (CAZymes), and dissimilatory sulfite reductase (*dsr*) genes.


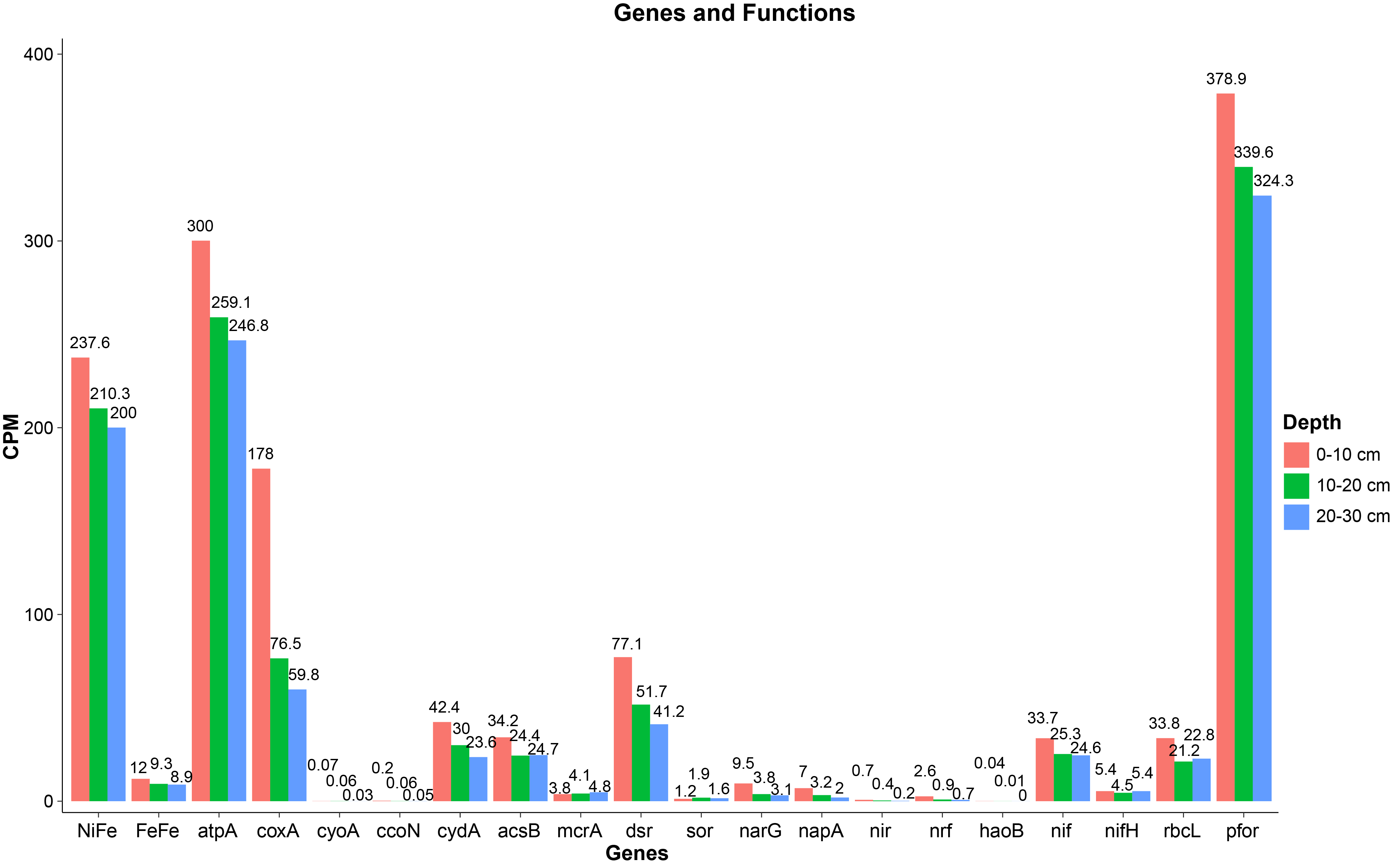
**Fig. S9** Read count of metagenomic reads for specific genes in different sediment depth. Read counts are standardized to CPM (count (read) per million reads). Abbreviation: NiFe/FeFe, [NiFe]-/[FeFe]-hydrogenases; *atpA*, ATP synthase; *coxA*, cytochrome c oxidase; *cyoA*, cytochrome o ubiquinol oxidase; *ccoN*, cytochrome c oxidase; *HCO*, haem-copper oxidase genes (*coxA*, *cyoA* and *ccoN*); *cydA*, cytochrome bd oxidase; *acsB*, acetyl-CoA synthase; *mcrA*, methyl-CoM reductase; *dsr*, dissimilatory sulfite reductase; *sor*, sulfur oxygenase/reductase; *narG*, dissimilatory nitrate reductase; *napA*, periplasmic nitrate reductase; *nir*, dissimilatory nitrite reductase; *nrf*, ammonifying nitrite reductase; *nif*, nitrogenase; *nifH*, nitrogenase iron protein; *rbcL*, ribulose 1,5-bisphosphate carboxylase; *pfor*, pyruvate-ferredoxin oxidoreductase.


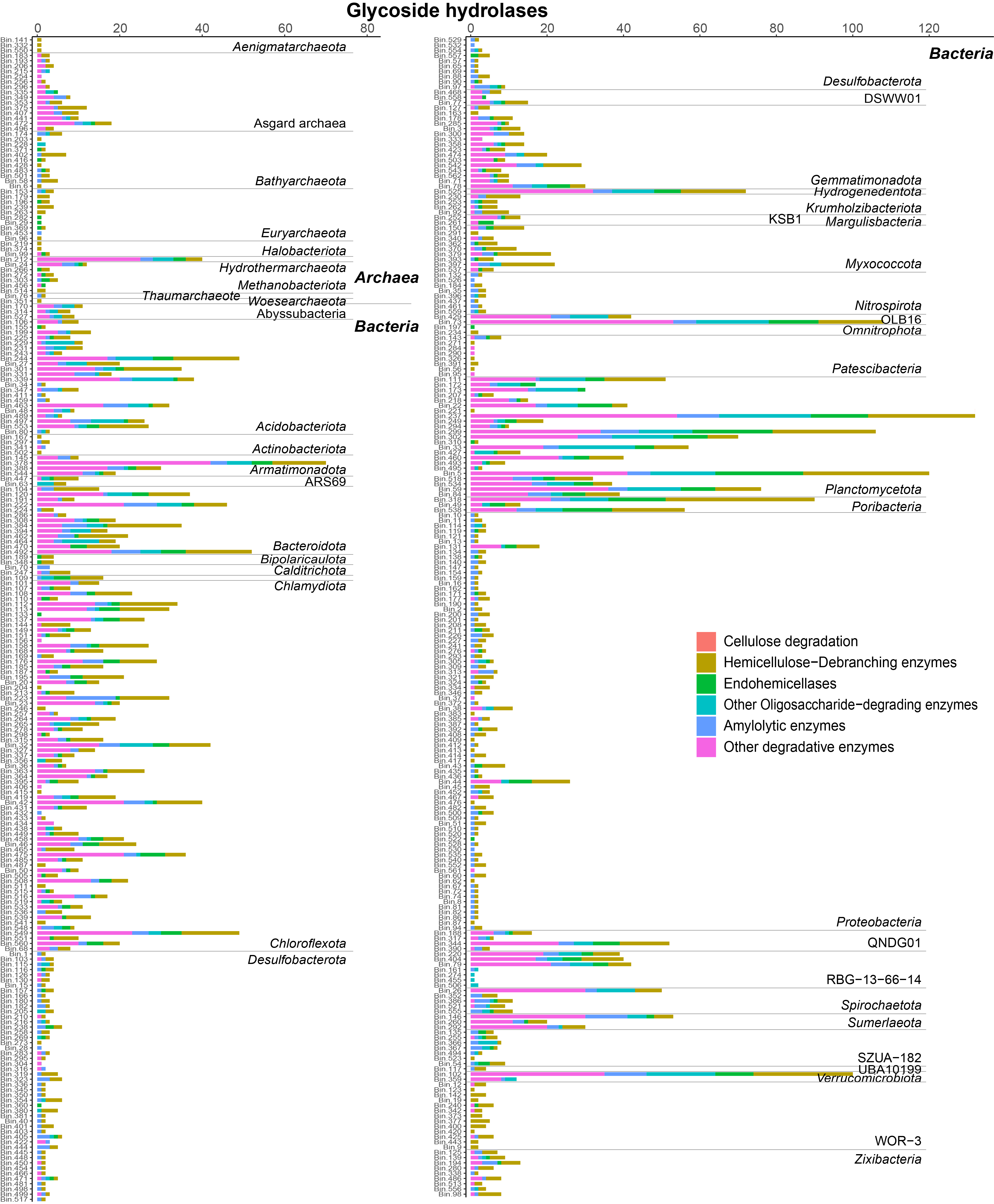


**Fig. S10** Glycoside hydrolases (GH) identified by CAZy searches of the MAGs. GH families that contain enzymes that are not specifically involved in degradation were specifically identified by Pfam or EC numbers in the annotations, based on Wrighton et al. 2014.


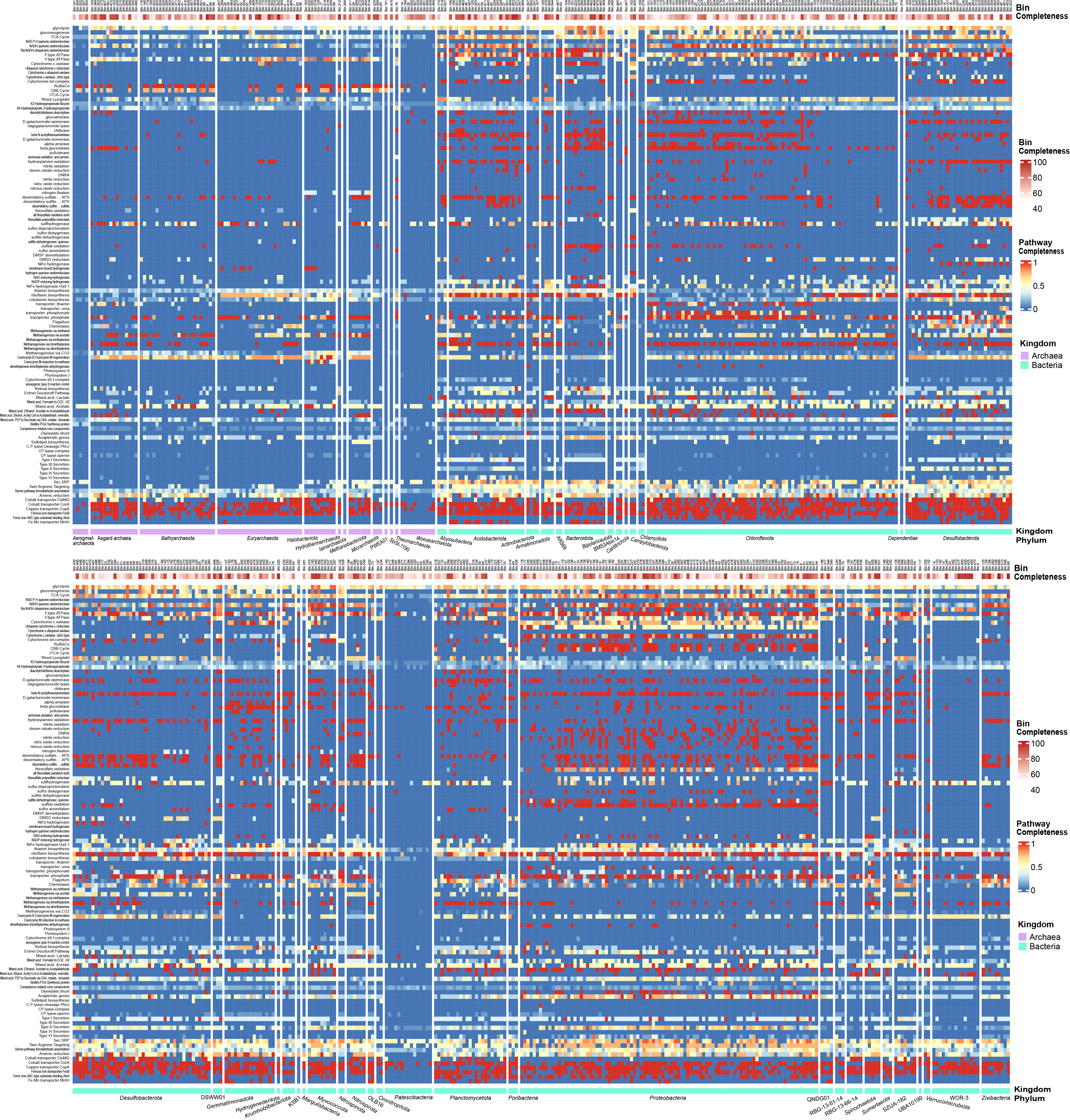
**Fig. S11** Pathway completeness of all refined MAGs calculated by KEGGDecoder. The pathway completeness is defined as the percentage of core genes of specific pathways identified in each MAG. Complete lists of metabolic genes or pathways can be found in Dataset S2 Sheet7. Detailed gene lists for each pathway indicated can be found at: https://github.com/bjtully/BioData/blob/master/KEGGDecoder/KOALA_definitions.txt. The up row of heatmap shows the MAG completeness, and the bottom raw represent the phylogenetic information of each MAG at phylum level (Archaea in purple and Bacteria in blue).


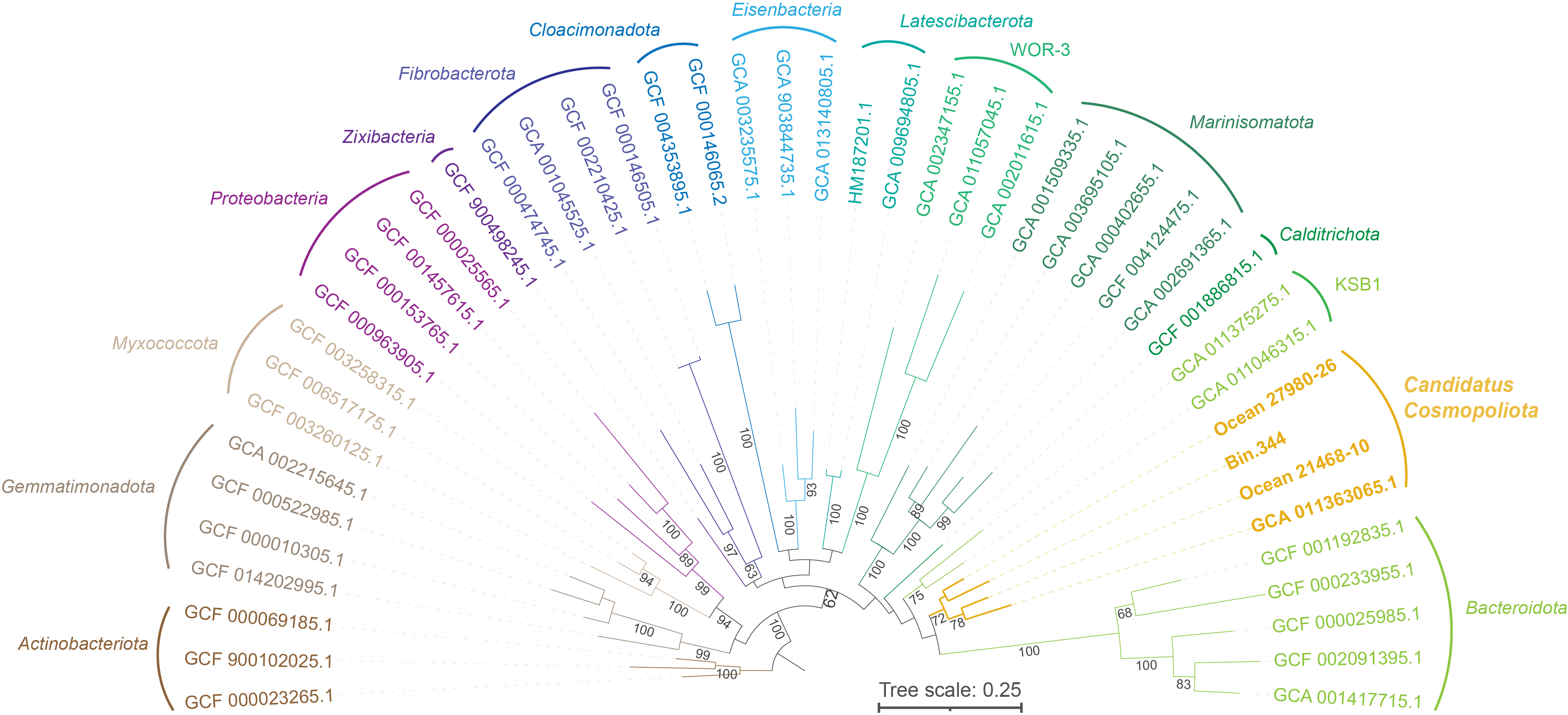
**Fig. S12** The phylogenetic tree of *Candidatus* Cosmopoliota and the adjacent phyla based on 16S rRNA genes. The genomes of *Ca.* Cosmopoliota are labeled in orange color and bold font.


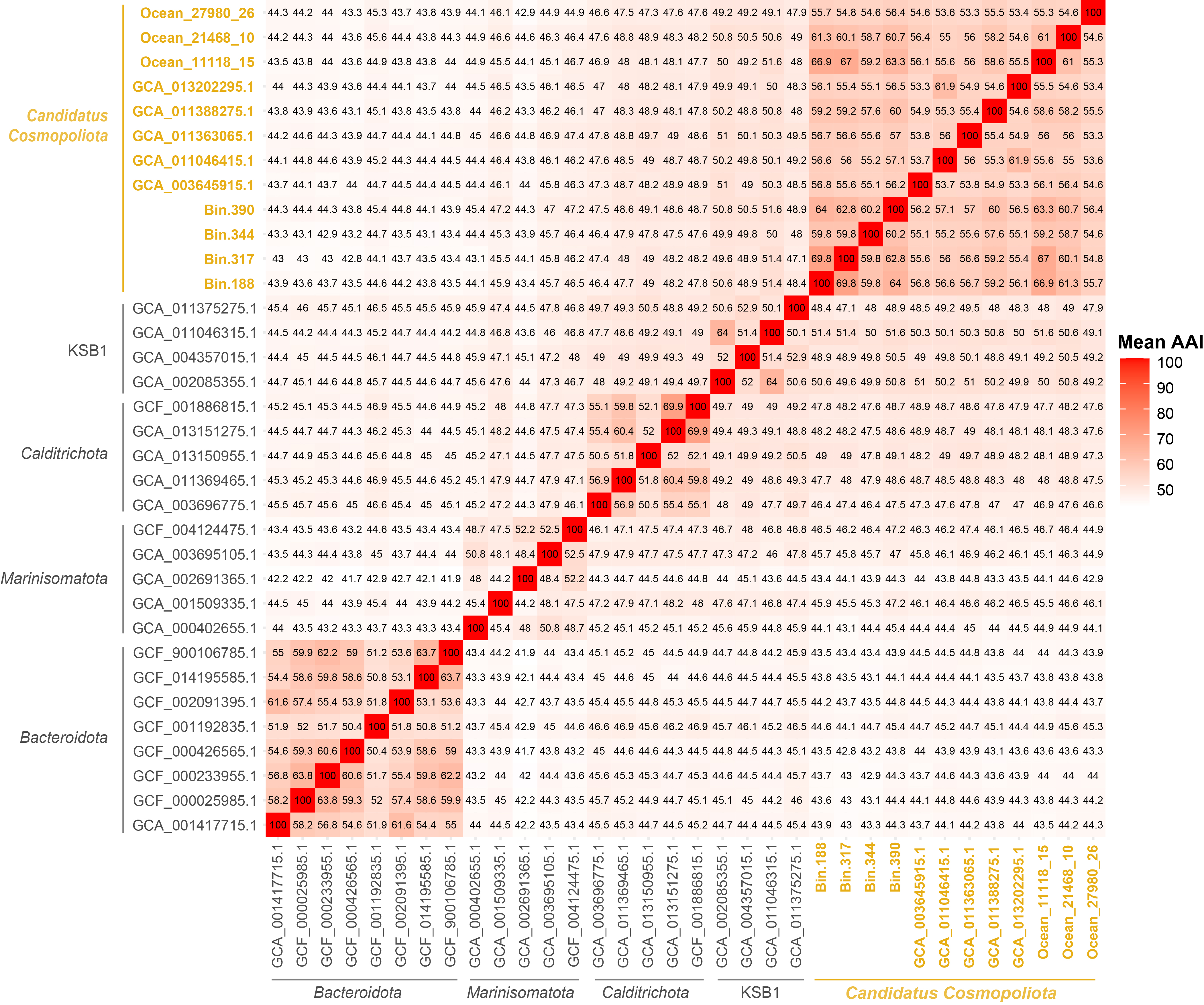
**Fig. S13** The average amino acid identity (AAI) values between each genome in *Candidatus* Cosmopoliota and the adjacent phyla. The genomes of *Ca.* Cosmopoliota are labeled in orange color and bold font.


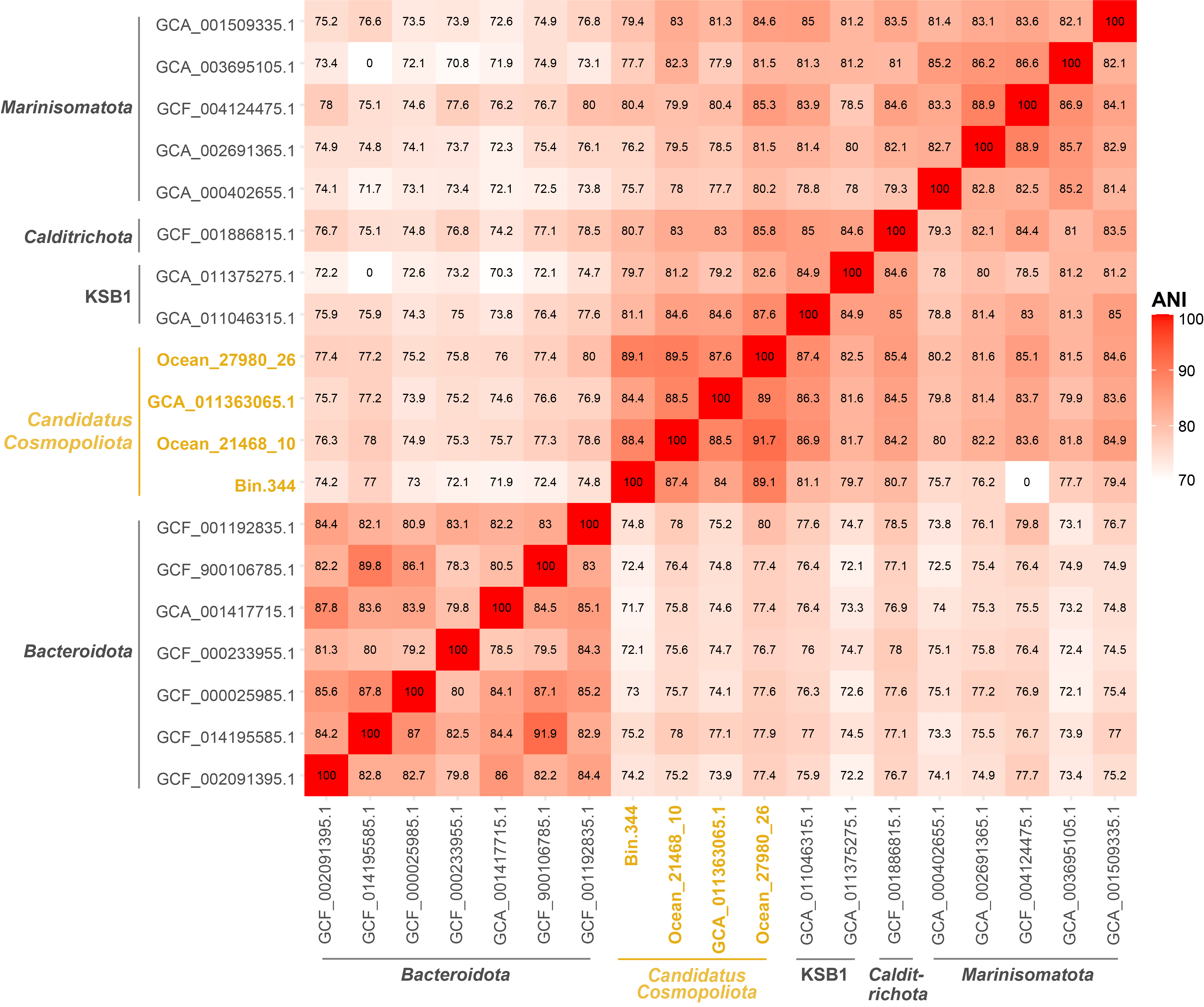
**Fig. S14** The average nucleotide identity (ANI) values between the 16S rRNA genes of *Ca.* Cosmopoliota and the adjacent phyla. The genomes of *Ca. Cosmopoliota* are labeled in orange color and bold font.
